# Salient brain entities labelled in *P2rx7*-EGFP reporter mouse embryos include the septum, roof plate glial specializations and circumventricular ependymal organs

**DOI:** 10.1007/s00429-020-02204-5

**Published:** 2021-01-11

**Authors:** Felipe Ortega, Rosa Gomez-Villafuertes, María Benito-León, Margaret Martínez de la Torre, Luis A. Olivos-Oré, Marina Arribas-Blazquez, María Victoria Gomez-Gaviro, Arturo Azcorra, Manuel Desco, Antonio R. Artalejo, Luis Puelles, María Teresa Miras-Portugal

**Affiliations:** 1 Departamento de Bioquímica y Biología Molecular, Facultad de Veterinaria, Universidad Complutense de Madrid, Madrid, Spain; 2 Instituto Universitario de Investigación en Neuroquímica (IUIN), Madrid, Spain; 3 Instituto de Investigación Sanitaria San Carlos (IdISSC), Madrid, Spain; 4 Department of Human Anatomy and IMIB-Arrixaca Institute, School of Medicine, Universidad de Murcia, Murcia, Spain; 5 Departamento de Farmacología y Toxicología, Facultad de Veterinaria, Universidad Complutense de Madrid, Madrid, Spain; 6 Departamento de Bioquímica, Instituto de Investigaciones Biomédicas “Alberto Sols”, Madrid, Spain; 7 Departamento de Bioingeniería e Ingenieria Aeroespacial, Universidad Carlos III de Madrid, Madrid, Spain; 8 Centro Nacional de Investigaciones Cardiovasculares (CNIC), Madrid, Spain; 9 Departamento de Ingeniería Telemática, Universidad Carlos III de Madrid, Leganés, Madrid, Spain; 10 IMDEA Networks Institute, Leganés, Madrid, Spain; 11 Medicina y Cirugía Experimental, Instituto de Investigación Sanitaria del Hospital Gregorio Marañón, Madrid, Spain; 12 Centro de Investigación Biomédica en Red de Salud Mental (CIBERSAM), Madrid, Spain

**Keywords:** P2X7 receptor, Purinergic system, Embryonic brain, *P2rx7*-EGFP mouse, Postarcuate organ, PArcO, Ventricular hypothalamic organ

## Abstract

The purinergic system is one of the oldest cell-to-cell communication mechanisms and exhibits relevant functions in the regulation of the central nervous system (CNS) development. Amongst the components of the purinergic system, the ionotropic P2X7 receptor (P2X7R) stands out as a potential regulator of brain pathology and physiology. Thus, P2X7R is known to regulate crucial aspects of neuronal cell biology, including axonal elongation, path-finding, synapse formation and neuroprotection. Moreover, P2X7R modulates neuroinflammation and is posed as a therapeutic target in inflammatory, oncogenic and degenerative disorders. However, the lack of reliable technical and pharmacological approaches to detect this receptor represents a major hurdle in its study. Here, we took advantage of the *P2rx7*-EGFP reporter mouse, which expresses enhanced green fluorescence protein (EGFP) immediately downstream of the *P2rx7* proximal promoter, to conduct a detailed study of its distribution. We performed a comprehensive analysis of the pattern of P2X7R expression in the brain of E18.5 mouse embryos revealing interesting areas within the CNS. Particularly, strong labelling was found in the septum, as well as along the entire neural roof plate zone of the brain, except chorioidal roof areas, but including specialized circumventricular roof formations, such as the subfornical and subcommissural organs (SFO; SCO). Moreover, our results reveal what seems a novel circumventricular organ, named by us postarcuate organ (PArcO). Furthermore, this study sheds light on the ongoing debate regarding the specific presence of P2X7R in neurons and may be of interest for the elucidation of additional roles of P2X7R in the idiosyncratic histologic development of the CNS and related systemic functions.

**Supplementary Information:**

The online version contains supplementary material available at 10.1007/s00429-020-02204-5.

## Introduction

The development of the mammalian CNS is a complex and dynamic process that requires an accurately orchestrated sequence of genetic, environmental, and biochemical events. The generation of neural cells, i.e. neurons, astrocytes and oligodendrocytes, implies a precise and positionally differential control of crucial processes, such as cell proliferation, cell fate determination, migration, maturation, synapse formation, network implementation, and eventually, controlled apoptosis, to define the correct neuronal number and location. The control of these processes accounts for multiple mechanisms including extracellular signaling molecules. Amongst such regulatory signals, extracellular ATP and other nucleotides are one of the more promising candidates in the regulation of CNS development (Zimmermann 2006).

Going beyond the classic metabolic function of ATP, which is associated to energy storage, its involvement in purinergic signaling acting as an extracellular transmitter/modulator, constitutes one of the oldest cell-to-cell communication systems evolved (Burnstock 1972; Burnstock et al. 1970; Oliveira et al. 2016). Signal transmission mediated by ATP begins with its loading into secretory vesicles, exerted by the vesicular nucleotide transporter (VNUT). This transporter was first characterized by its pharmacological and biochemical properties (Bankston and Guidotti 1996; Gualix et al. 1996, 1999), and finally was cloned in 2008 (Sawada et al. 2008), hence enabling a more detailed study of its distribution in the CNS. VNUT exhibits important expression levels in human and mouse brains (Menendez-Mendez et al. 2017; Sawada et al. 2008), and it has been shown to modulate various physiological and pathological processes in the nervous system (Menendez-Mendez et al. 2015) (for a review see Miras-Portugal et al. 2019a). Once released to the extracellular space, ATP and other nucleotides interact with cell-surface purinoceptors (P2 receptors) (Burnstock et al. 2011), which have been classified into two subfamilies, the P2X and P2Y receptors (Burnstock 2007b). P2X receptors are composed of three subunits taken among seven available subtypes (P2X1-7), either as homo- or heterotrimers, forming ligand-gated ion channels permeable to Na^+^, K^+^ and Ca^2+^. In contrast, P2Y receptors are a subfamily of eight G-coupled metabotropic receptors present in mammals (P2Y_1,2,4,6,11,12,13,14_) (Burnstock 2007b; Burnstock et al. 2011). In the CNS, purinergic receptors regulate cell growth and migration during development, and as the CNS matures also modulate glia–glia/neuron-glia interactions, mechanosensory transduction, and autonomic functions (Abbracchio et al. 2009; Burnstock 2007a).

Amongst the P2 receptors, P2X7R constitutes a promising target in the regulation of brain physiology and pathophysiology (Del Puerto et al. 2013; Miras-Portugal et al. 2016, 2017). P2X7R is characterized by its low affinity for ATP, and the expression of several splice variants in the nervous system. It exhibits a long C-terminal domain that could interact with different intracellular proteins and channel dilatation mechanisms (Miras-Portugal et al. 2017; Sperlagh and Illes 2014; Virginio et al. 1999). Signaling mediated by P2X7R is known to regulate crucial aspects of neuronal cell biology before and after CNS maturation. For instance, P2X7R is a key factor in the regulation of axonal elongation, path-finding and synapse formation during the maturation of hippocampal neurons (Del Puerto et al. 2012; Diaz-Hernandez et al. 2008). Additionally, P2X7R exerts a neuroprotective effect against glutamate-mediated excitotoxicity and neurotrophic deprivation during the differentiation of cerebellar granule neurons (Miras-Portugal et al. 2019b; Ortega et al. 2009, 2011, 2010; Queipo et al. 2017). Furthermore, in the adult and aged CNS, P2X7R regulates neurotransmitter release, and behaves as an active modulator of neuroinflammation, constituting an emergent therapeutic target in the field of inflammatory, oncogenic and degenerative neural disorders (Burnstock and Knight 2018; Gomez-Villafuertes et al. 2009, 2015; Miras-Portugal et al. 2016, 2017). However, the lack of specific and reliable technical and pharmacological approaches to detect the receptor has classically been a major hurdle in the study of purinergic receptors and, in particular, of P2X7R.

**Fig. 1:**
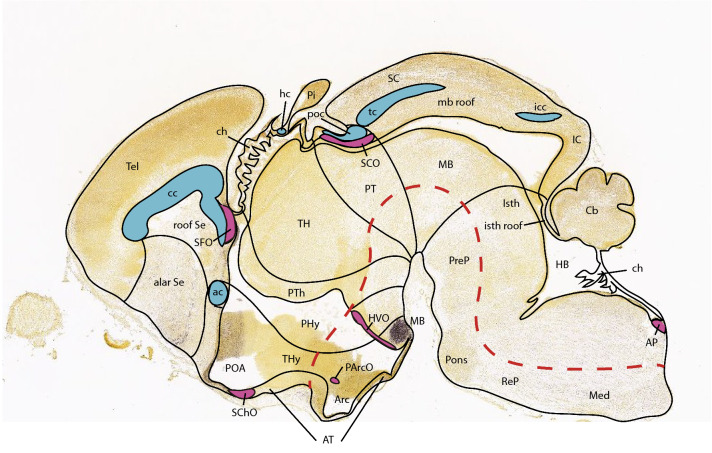
Semischematic tracing on top of a real E18.5 sagittal section (extracted from the Allen Developing Mouse Brain Atlas) of anatomic details of the brain roof plate including diverse identified roof commissures (blue), chorioidal tela roof patches (ch), and the main circumventricular organs (violet)

Transgenic mice expressing fluorescent proteins under the control of specific promoters of purinergic receptors are tools that facilitate the identification of the expression patterns of individual receptors during development (Zimmermann 2006). We used in this work the *P2rx7*-EGFP reporter mouse, which expresses EGFP immediately downstream of the mouse *P2rx7* proximal promoter (Gong et al. 2003), allowing a detailed study of its tissue distribution in the CNS. We show a comprehensive analysis of the pattern of expression of P2X7R in the brain of E18.5 mouse embryos. Neuronal expression was quite selective topographically. Strongest expression was found in the septum, as well as along the entire neural roof plate (rp) zone of the brain, except chorioidal roof areas, but including specialized circumventricular roof formations, such as the SFO and the SCO (Fig. 1). Distinct *P2rx7*-EGFP signal was also observed at some other non-median circumventricular organs or ependymal specializations, and some fiber tracts were selectively visualized, apart from various neuronal populations. Considering its reported role on axonal guidance and neuronal differentiation, as well as in glial functions, this analysis may be of interest for the elucidation of additional roles of P2X7R in the idiosyncratic histologic development of the CNS and related systemic functions.

## Materials and methods

### Animals

All animal procedures were carried out at the Universidad Complutense de Madrid in accordance with European and Spanish regulations (2010/63/EU; RD 53/2013), following the guidelines of the International Council for the Laboratory Animal Science and with the approval of the institutional Animal Care and Use Committee (PROEX-286/15). *P2rx7*-EGFP reporter mice were obtained from the United States National Institutes of Health (Mutant Mouse Regional Resource Center; stock 011959-UCD) (Gong et al. 2003), with kind permission of Dr. M. Nedergaard (University of Rochester Medical School, Rochester, NY). These transgenic mice contain multiple copies of a modified bacterial artificial chromosome (BAC) in which the EGFP reporter gene is inserted immediately upstream of the *P2rx7* gene coding sequence.

### Retrotranscription and quantitative real-time PCR

Total RNA was extracted from E9.5 (*n* = 5), E13.5 (*n* = 10), and E18.5 (*n* = 5) embryonic brains from *P2rx7*-EGFP reporter mice using the Speedtools total RNA extraction kit (Biotools, Madrid, Spain), following the manufacturer’s instructions. After digestion with TURBO DNase (Thermo Fisher Scientific; Waltham, MA), total RNA was quantified with a Nanodrop One spectrophotometer (Thermo Fisher Scientific), and 1 µg/mL of RNA was reverse-transcribed, using the M-MLV reverse transcriptase, 6 µg of random primers and 350 µM dNTPs (Thermo Fisher Scientific). Quantitative real-time PCR reactions were carried out using LuminoCt^®^ qPCR Readymix (Sigma, Madrid, Spain), 5 µL of the reverse-transcribed product, commercial gene-specific primers, and TaqMan MGB probes for mouse P2X7 sequence (Thermo Fisher Scientific). Specific primers and a probe were designed for EGFP amplification: forward primer 5′-gaagcgcgatcacatggt-3′, reverse primer 5′-ccatgccgagagtgatcc-3′ (both from Sigma), MGB probe 5′-tgctggag-3′ (Roche Diagnostics, Barcelona, Spain). Fast thermal cycling was performed using a StepOnePlus™ Real-Time PCR System (Thermo Fisher Scientific) as follows: denaturation, one cycle of 95 °C for 20 s, followed by 40 cycles of 95 °C for 1 s and 60 °C for 20 s each. The relative standard curve method was used to evaluate the relative expression levels of both EGFP and P2X7 transcripts. Standard curves were obtained from dilutions of a cDNA sample prepared from mouse brain total RNA, and unknown sample quantitative values were interpolated from the appropriate standard curve.

### Western blotting

E9.5 (*n* = 4), E13.5 (*n* = 4), and E18.5 (*n* = 4) embryonic brains from *P2rx7*-EGFP reporter mice were lysed and homogenized for 1 h at 4 °C in lysis buffer containing 50 mM Tris–HCl, 150 mM NaCl, 1% Nonidet P40, Complete™ Protease Inhibitor Cocktail Tablets (Roche Diagnostics), 1 mM sodium orthovanadate (Sigma) and 1.5 µM okadaic acid (Merck Life Science, Madrid, Spain), pH 7.4. Protein extracts (20 µg) were electrophoresed on a 10% Tris–Glycine SDS-PAGE gel and transferred to nitrocellulose membranes (Amersham GE, Barcelona, Spain) saturated with 5% non-fat dried milk or 3% BSA for 1 h at RT. Blots were incubated overnight at 4 °C with the following antisera: rabbit anti-P2X7 (1:1000, 70 KDa; Alomone labs, Jerusalem, Israel), rabbit anti-GFP (1:1000, 27 KDa; Thermo Fisher Scientific), and rabbit anti-GAPDH (1:10,000, 37 KDa; Sigma). Then, the blots were washed in PBS-Tween and incubated for 1 h at RT with goat anti-rabbit IgGs coupled to horseradish peroxidase at 1:5000 dilution (Dako Cytomation, Glostrup, Denmark). Proteins were visualized by enhanced chemoluminescence detection (Perkin Elmer, Houston, TX). Images were captured with an ImageQuant LAS 500 device (Amersham GE) and analysed using ImageQuant software (Amersham GE).

### Immunostaining

*P2rx7*-EGFP embryonic brains (E14.5 or E18.5) were fixed for 3 h in 4% paraformaldehyde. For production of free-floating vibratome sections, brains were embedded in 4% agarose solution, and serial sections were cut 100 µm-thick in sagittal or horizontal section planes (our horizontal plane was parallel to the optic tract, i.e., horizontal to the hypothalamus, diencephalon and rostral midbrain regions). Primary antibodies—chicken anti-GFP (1:500; Aves Labs, Davis, CA) and rabbit anti-P2X7 (1:100; Alomone Labs, Israel) were diluted in 0.1 M PBS containing 0.5% Triton X-100 (wt/vol.) and 2% BSA (wt/vol.). Secondary antibodies used were anti-Chicken Alexa Fluor 488 (1:400; Thermo Fisher Scientific) and anti-rabbit Alexa Fluor 546 (1:500; Thermo Fisher Scientific), and the sections were counterstained with DAPI (Sigma). This material was interpreted morphologically according to standard atlases of the mouse brain and the prosomeric morphologic brain model (Puelles et al. 2013; Puelles and Rubenstein 2003, 2015). All images were acquired on a Leica TCS SPE confocal microscope using the 5x, 10x, 40x and 63x W/IR objectives.

## Results

**Fig. 2:**
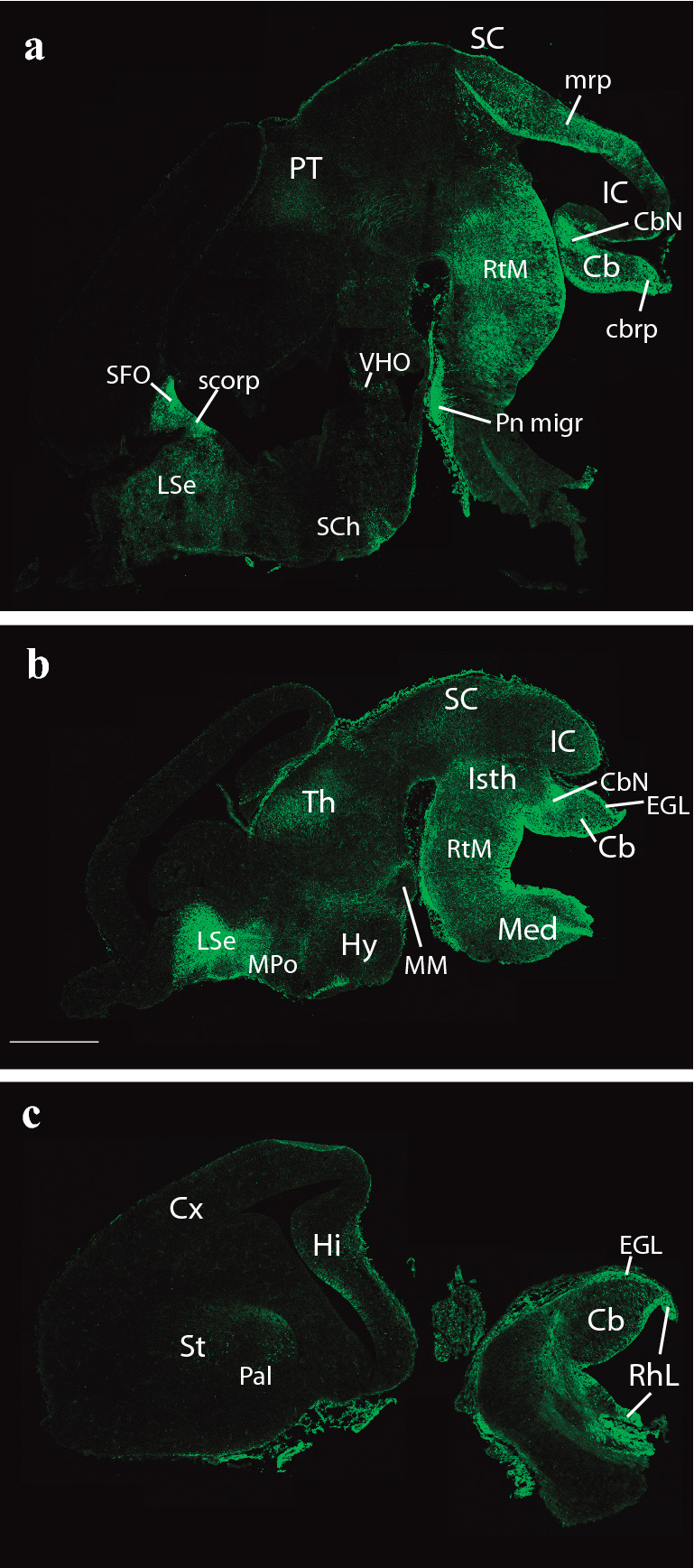
Expression of the P2X7 receptor and EGFP reporter on *P2rx7*-EGFP mice in three sagittal sections at E14.5 (**a, b, c**). Scale bar represents 1 mm

**Fig. 3:**
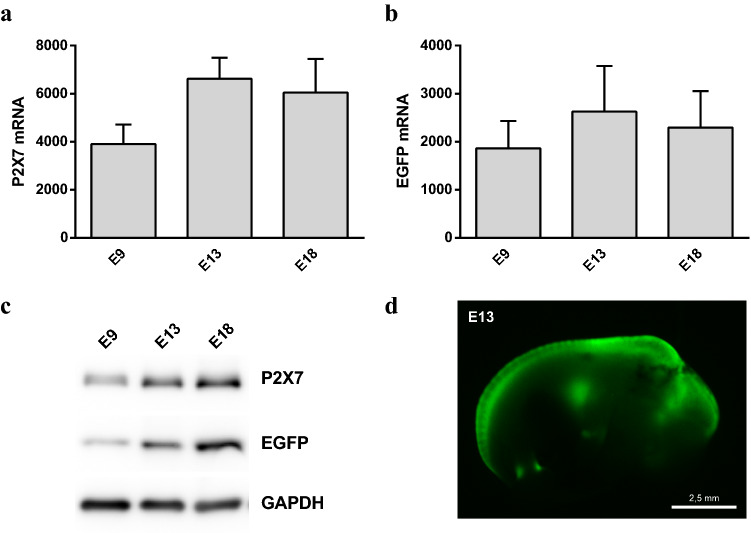
Expression of the P2X7R and EGFP reporter on *P2rx7*-EGFP mice. **a** Analysis of the mRNA levels of P2X7R at E9, E13 and E18 brains showing that the mRNA is present at early stages of CNS development. **b** Analysis of the mRNA levels of EGFP reporter gene at E9, E13 and E18 brains. **c** Analysis of the protein expression of P2X7R (~ 68 KDa) and its associated reporter EGFP (~ 25 KDa) at E9, E13 and E18 brains. The expression of the constitutively expressed protein GAPDH was used as a loading control (~ 37 KDa). Note that both P2X7R and EGFP are already detectible at embrE9, increasing subsequently at E13 and E18. **d** Image depicting an EGFP^+^ E13 embryo, highlighting the expression of the reporter in the developing CNS as well as in the heart. Scale bar represents 2.5 mm

Prior to offering an exhaustive analysis of E18.5 embryonic *P2xr7*-EGFP reporter mice, we confirmed that the expression of P2X7R is readily detectable at early stages of CNS development (e.g., at E14.5; Fig. 2). P2X7R transcript was already present at E9 brains of *P2xr7*-EGFP mice and increased at later stages (Fig. 3).

As expected, the mRNA levels of the receptor matched the expression observed for the EGFP reporter transcript (Fig. 3). Furthermore, the correlation between mRNA and protein levels of P2X7R, as well as their associated reporter EGFP, was also confirmed by western blot experiments, positive results being perceptible at embryonic day 9 and showing a significant presence at the stages selected for the present study (E18.5) (Fig. 3). In addition, we further confirmed the specificity of this correlation by immunohistochemical assays (Suppl. Figure 1).

Two remarkable and unexpected results of the distribution of *P2rx7*-EGFP observed in the mouse brain were its clear-cut localization to non-chorioidal parts of the entire brain rp, as well as to a number of circumventricular organs. Various neuronal populations and tracts also expressed selectively this marker. For the sake of unitary conception, in the following sections, we will address first the rp-associated expression domain throughout the brain, and second, the observed circumventricular organs. Finally, we will cover systematically other heterogeneous results obtained in different brain territories (telencephalon (Tel), hypothalamus (Hy), diencephalon, midbrain (MB), hindbrain and spinal cord).

### The brain roof plate and expression of *P2rx7*-EGFP

**Fig. 4:**
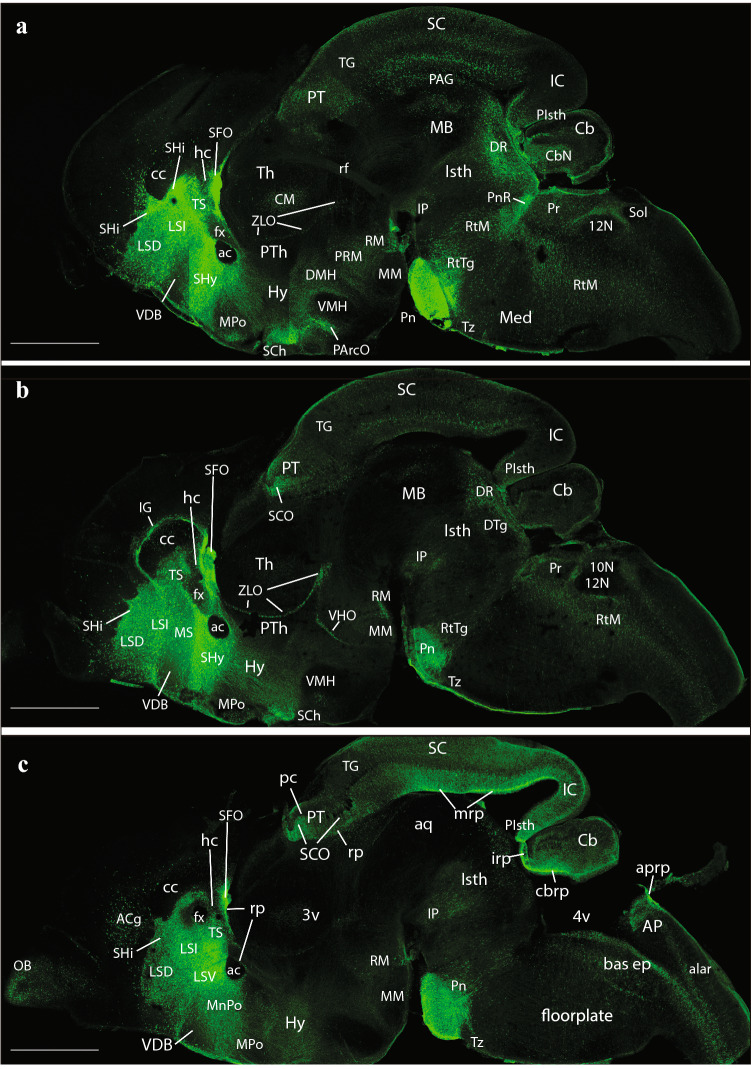
Expression of the *P2rx7*-EGFP signal at stage E18.5. **a–c** Series of sagittal sections depicting identifiable signal of the reporter along the different areas highlighted in the results part (see also Abbreviations). Scale bar represents 1 mm

**Fig. 5:**
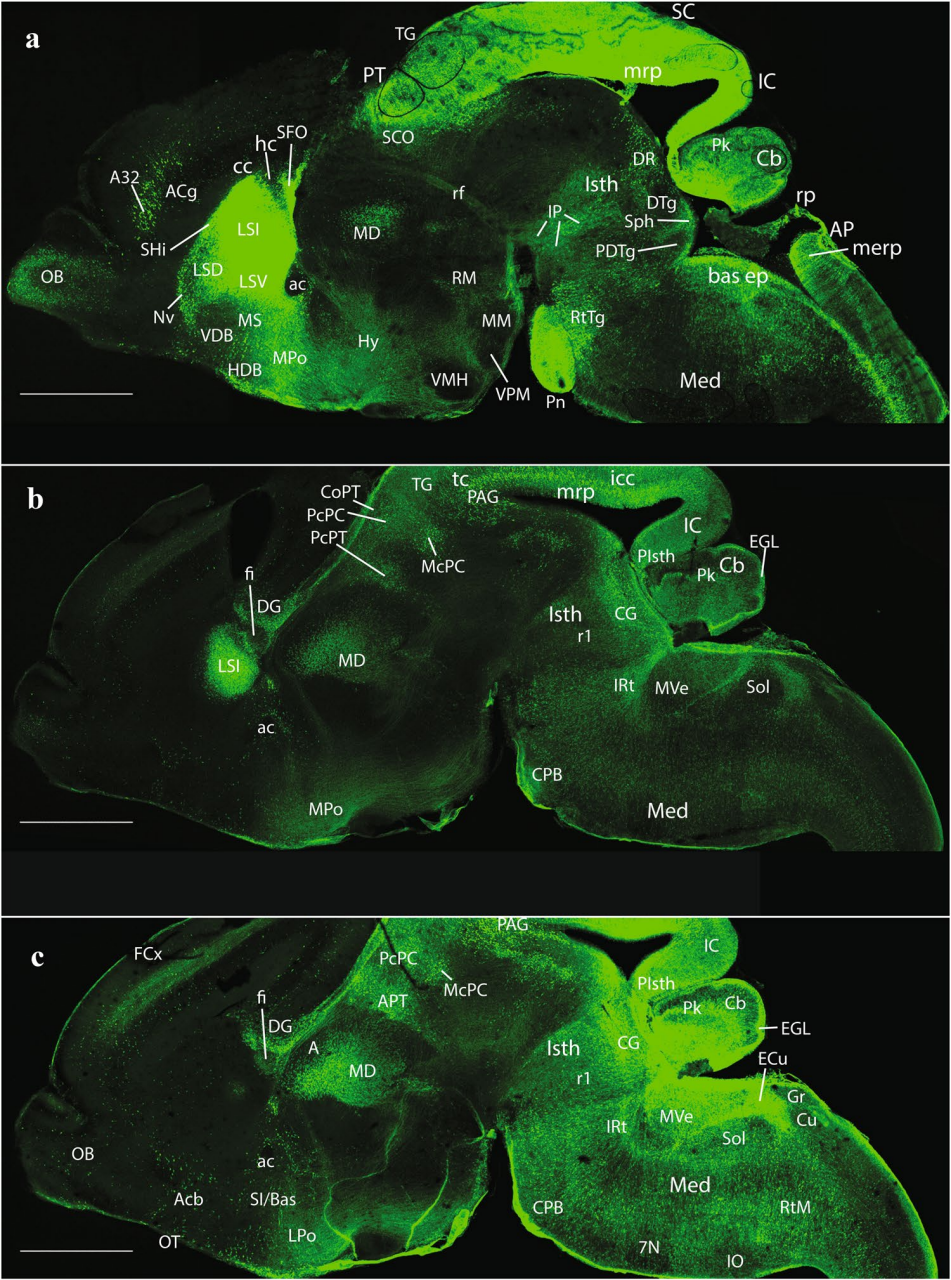
Expression of the *P2rx7*-EGFP signal at stage E18.5. **a–c** Continuation of the series of sagittal sections depicting an identifiable signal of the reporter along the different areas highlighted in the results part (see also Abbreviations). Scale bar represents 1 mm

**Fig. 6:**
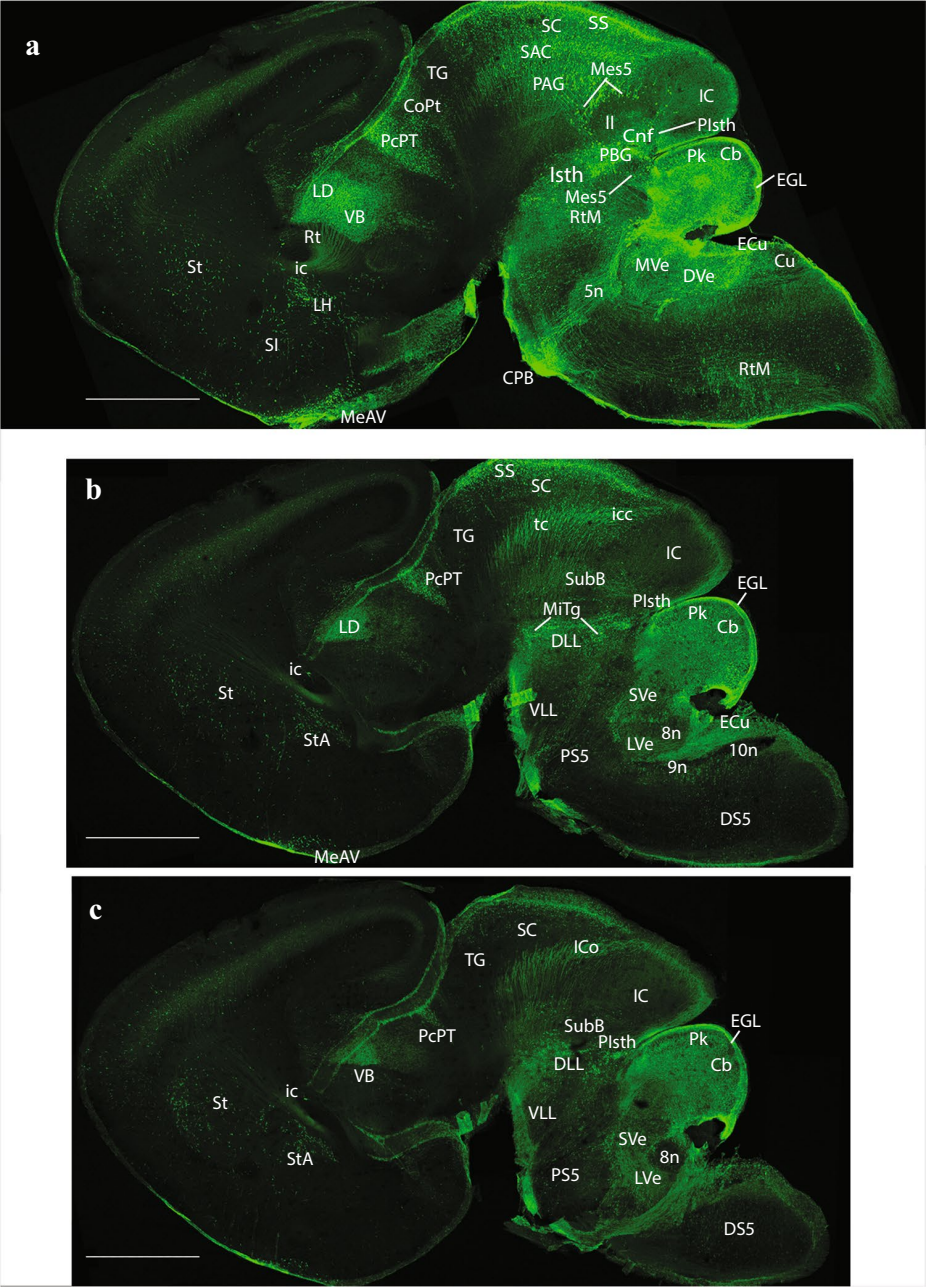
Expression of the *P2rx7*-EGFP signal at stage E18.5. **a–c** Continuation of the series of sagittal sections depicting identifiable signal of the reporter along the different areas highlighted in the results part (see also Abbreviations). Scale bar represents 1 mm

**Fig. 7:**
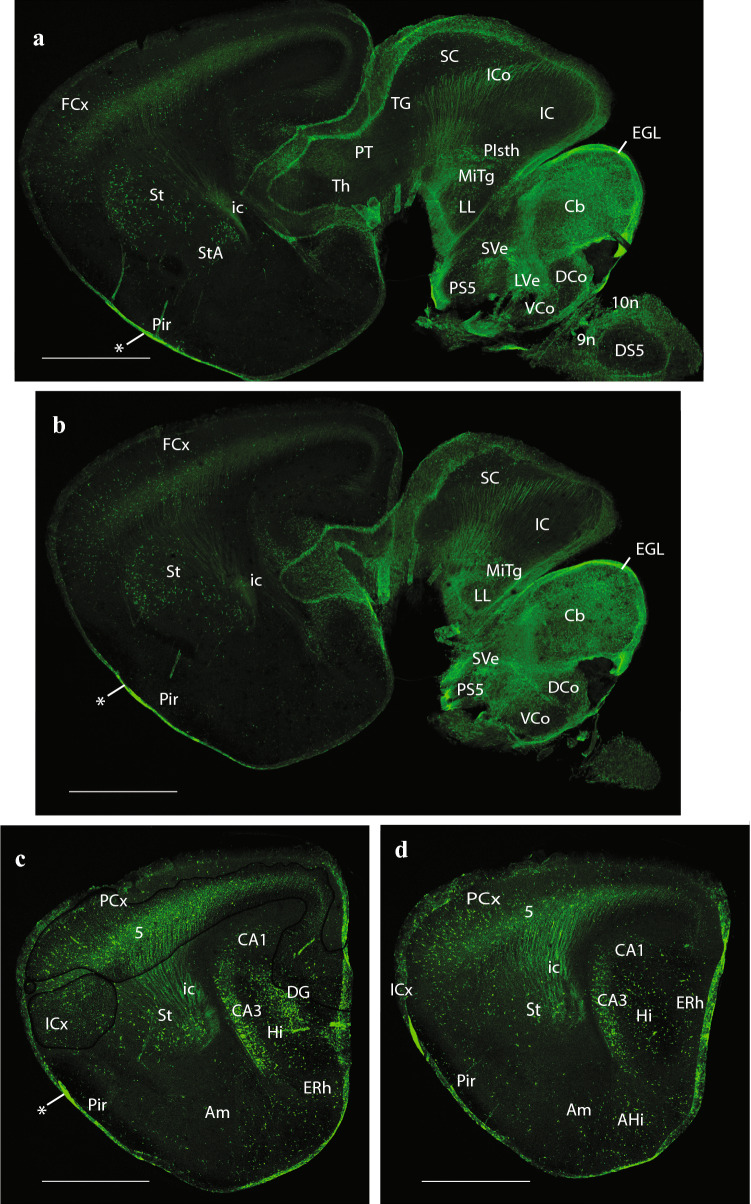
Expression of the *P2rx7*-EGFP signal at stage E18.5. **a–d** Final series of sagittal sections depicting identifiable signal of the reporter along the different areas highlighted in the results part (see also Abbreviations). Asterisk in **a–c** indicates labelling possibly corresponding to glial endfeet of radial glia cells and/or free astrocytes, where these contact the pial basal membrane. Scale bar represents 1 mm

**Fig. 8:**
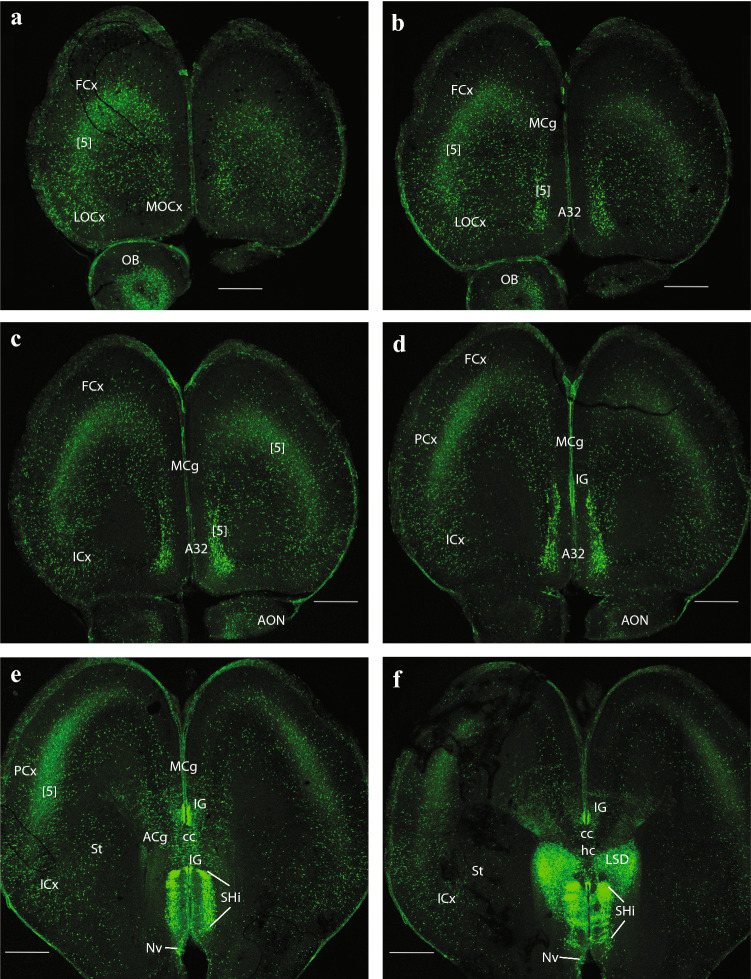
Expression of the *P2rx7*-EGFP signal at stage E18.5. **a–f** Rostrocaudal series of horizontal sections depicting identifiable signal of the reporter along the different areas highlighted in the results part (see also Abbreviations). This mapping is based on the recently updated and expanded prosomeric model of the forebrain. Scale bar represents 500 µm

**Fig. 9:**
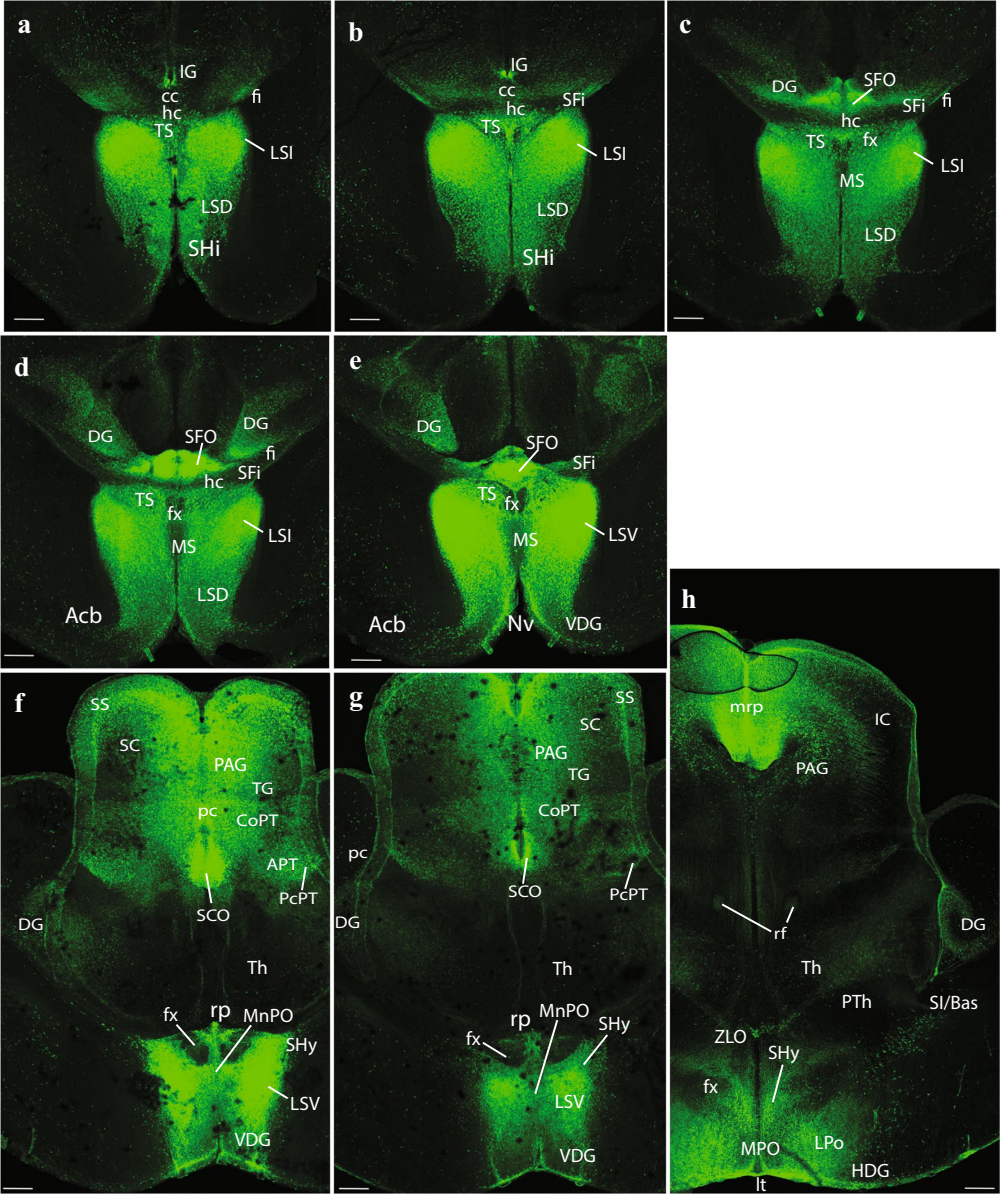
Expression of the *P2rx7*-EGFP signal at stage E18.5. **a–h** Continuation of the rostrocaudal series of horizontal sections depicting identifiable signal of the reporter along the different areas highlighted in the results part (see also Abbreviations). This mapping is based on the recently updated and expanded prosomeric model of the forebrain. Scale bar represents 500 µm

**Fig. 10:**
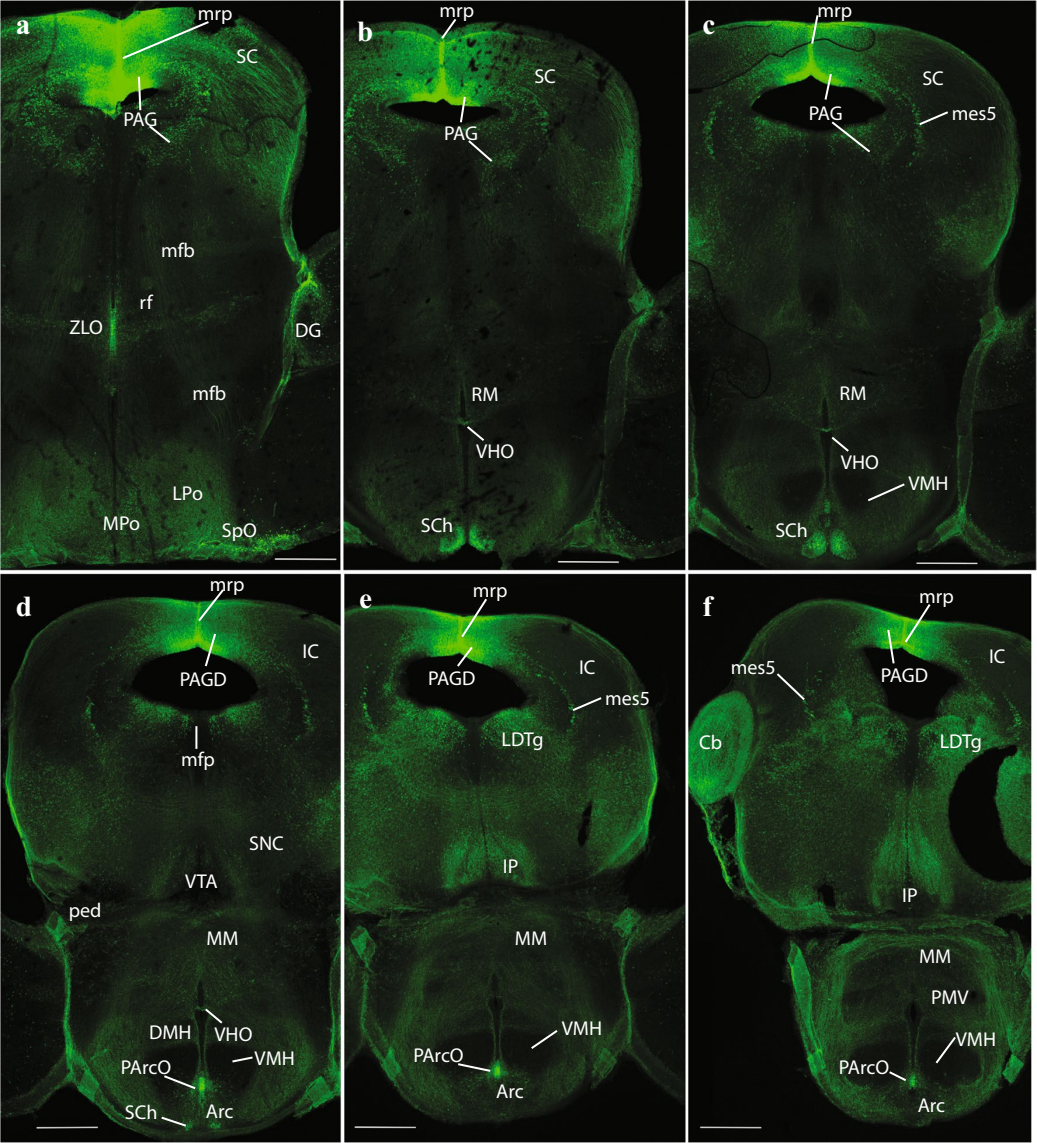
Expression of the *P2rx7*-EGFP signal at stage E18.5. **a–f** Continuation of the rostrocaudal series of horizontal sections depicting identifiable signal of the reporter along the different areas highlighted in the results part (see also Abbreviations). This mapping is based on the recently updated and expanded prosomeric model of the forebrain. Scale bar represents 500 µm

**Fig. 11:**
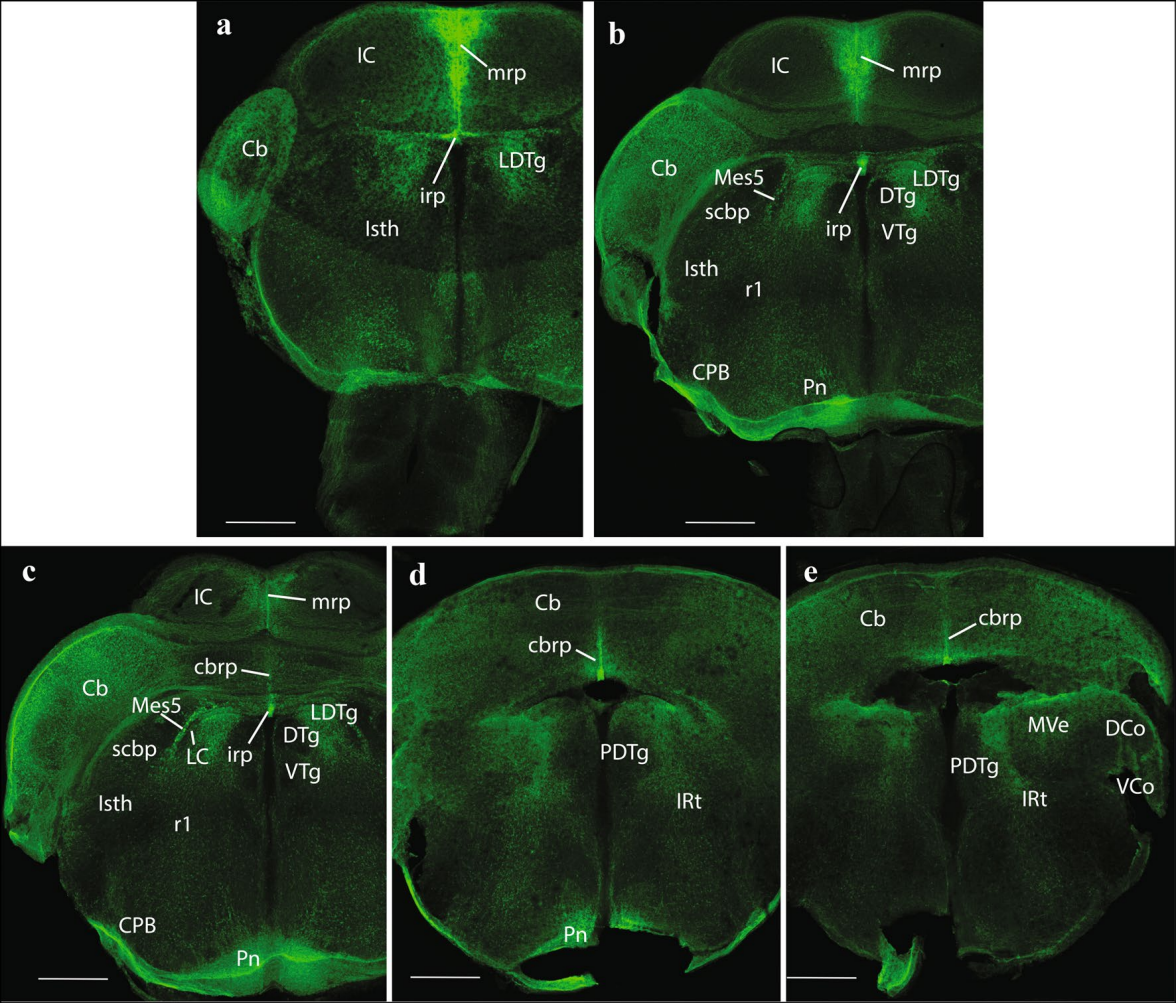
Expression of the *P2rx7*-EGFP signal at stage E18.5. **a–e** Continuation of the rostrocaudal series of horizontal sections depicting identifiable signal of the reporter along the different areas highlighted in the results part (see also Abbreviations). This mapping is based on the recently updated and expanded prosomeric model of the forebrain. Scale bar represents 500 µm

**Fig. 12:**
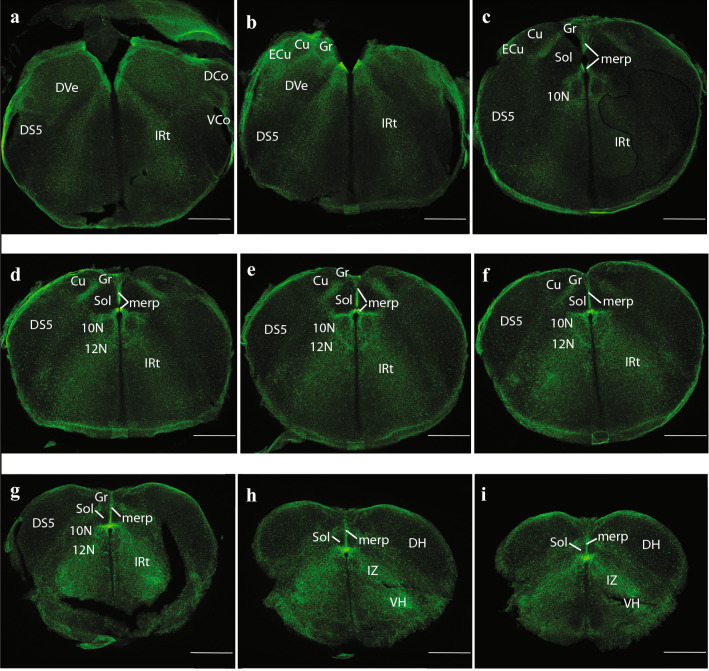
Expression of the P2rx7-EGFP signal at stage E18.5. **a–i** Final rostrocaudal series of horizontal sections depicting identifiable signal of the reporter along the different areas highlighted in the results part (see also Abbreviations). This mapping is based on the recently updated and expanded prosomeric model of the forebrain. Scale bar represents 500 µm

At E14.5, medial sagittal sections showed strong EGFP labeling of the septocommissural rp (Scorp), including the anlage of the SFO (Scorp; SFO; Fig. 2a, b), as well as the midbrain roof plate (mrp), cerebellar roof plate (cbrp), and the pontine migration (Pn migr) stream (mrp; cbrp; Pn migr Fig. 2a, b). Labeled ependymal cells were distinguished at the circumventricular periventricular hypothalamic organ (VHO; Fig. 2a, b). A more lateral section (Fig. 2b, c) shows massive labeling of the lateral septal mantle (LSe), a bundle of labeled fibres within the thalamus (Th), plus strong labeling at the cerebellar nuclei primordia (CbN) and the cerebellar external granular cell layer (EGL), as well as a field of labeled radial glial processes crossing the hindbrain reticular nucleus (Rt). More laterally, the telencephalic ganglionic eminences display labeled cell populations within the striatum (St) and pallidum (Pal) mantle primordia (St; Pal; Fig. 2b, c). Figure 1 illustrates a nearly median sagittal section through an E18.5 mouse brain, in which we have marked a number of details relative to rp commissures (in blue), chorioidal tela (ch) roof patches, and circumventricular organs (violet), apart from the main forebrain, midbrain and hindbrain anatomic regions, which will be useful for reference in the following description of E18.5 results. We illustrate to this end representative sections from two E18.5 mouse brains, one sagittal (Figs. 4, 5, 6, 7), and the other horizontal (Figs. 8, 9, 10, 11, 12). Our anatomic interpretations were aided by other similarly processed brains counterstained with red fluorescent immunoreaction for either tyrosine hydroxylase (TH) or calbindin (Calb1) (not shown). These markers characterize well-known neuronal cell populations, tracts or neuropiles, which served as neural landmarks.

The first two sagittal sections illustrate *P2rx7*-EGFP signal close to, or at, the telencephalic (septocommissural) roof midline. The corpus callosum (cc), hippocampal commissure (hc) and anterior commissure (ac), as well as the fornix tract (fx) are identifiable as non-fluorescent packets of sectioned fibers (Fig. 4a, b). Strong fluorescent signal for the marker is observed at the circumventricular SFO (SFO; Figs. 4a–c, 5a, 9c–e, (Ganong 1977)). Other septal, cortical or preoptic sites of expression distinguished in the same images (septohippocampal nucleus (SHi), tectal gray (TG), indusium griseum (IG), triangular septal nucleus (TS), lateral dorsal nucleus (LSD), lateral intermediate septal nucleus (LSI), medial spetal (MS), median preoptic nucleus (MnPo)) do not belong strictly to the rp, but to adjacent alar-plate-derived tissue (see below separate description under ‘telencephalon’). The next rp portion visible is at the pretectum (PT; caudal diencephalon; the pineal thalamic roof was torn away during dissection of the brain, a frequent artifact). There appears a strong *P2rx7*-EGFP signal at the ependymal thickening forming the pretectal SCO (SCO; Figs. 4b, c, 5a, 9f, g). This organ is covered by the negative packets of posterior commissure fibers (pc; Figs. 4c, 9f, g). In the next sagittal section, the pc appears traversed radially by many fluorescent glial cell processes, which represent the astroglial median palisade of the pretectal rp proper (note fibrillary labelling between the PT and SCO tags; Fig. 5a; unclear in Fig. 9f, g due to excess of signal).

Caudally to the pretectal roof the mrp also appears strongly fluorescent precisely at the midline (mrp; Figs. 4c, 5a, b, 9 h, 10a–f, 11a–c; note the roof glial palisade at the inferior colliculus (IC), which fans out bilaterally as it approaches the pial surface; IC; mrp; Figs. 11a, b). A fibrillar radial arrangement of labelled glial processes covered by the tectal and intercollicular commissures (tc, icc) can be observed in Fig. 5b. As found in the septum, some paramedian alar midbrain structures appear labeled at levels through the superior colliculus (SC), outside the local rp palisade, as can be corroborated at both sides of it in sagittal and horizontal sections (SC; mrp; Figs. 5c, 9 h, 10a–f).

The strongly labeled median glial palisade of the rp likewise extends into the isthmus (Isth) (Isthmic roof plate (irp); Isth; Figs. 4c, 5a, 11a–c) and the cerebellum (Cb) (cbrp; CB; Figs. 4c; 9c–e), and then jumps from there into the area postrema (AP) and associated caudal medullary roof plate (merp) (Area posrtrema roof plate (aprp); merp; AP; Figs. 4c, 5a, 11c–e, 12c–i). Note fibrillary palisade arrangement of labelled medullary roof glia cells in Fig. 5a, which also extends less massively into the spinal roof, there appears also weak signal at the hindbrain chorioidal rp (rp; Fig. 5a, between the Cb and AP tags).

### Expression of *P2rx7*-EGFP at circumventricular ependymal specializations

We observed that *P2rx7*-EGFP signal appears distinctly at sites with specialized ependym, which sometimes correspond to recognized circumventricular organs. Some of these specializations occur at the rp. The most evident examples are the *SFO* (Ganong 1977) and the *SCO* (Grondona et al. 2012; Munoz et al. 2019) already described in the previous section on the rp (SFO; Figs. 4a–c, 5a, 9c–e, SCO; Figs. 4b, c, 5a, 9f,g).

Other specializations occur in the lateral brain wall, appearing as linear fluorescent ependymal sites that extend along given known interneuromeric limiting landmarks or other sorts of limits, in either alar or basal plate regions. One limit-associated site is formed by the ependymal bodies of the glial palisade limiting the Th from the prethalamus (PTh) (the so-called zona limitans interthalamica), a thin transverse ventricular ridge extending between the alar-basal boundary and the rp at the cited boundary. This locus has been identified as an embryonic secondary organizer (also known as mid-diencephalic organizer; review in (Puelles and Martinez 2013)), where SHH, WNT3a and WNT8b signals are released and influence the fates of both prethalamic and thalamic territories. It is also possible that these or other signals from the zona limitans guide fiber tracts that cross this boundary orthogonally, as occurs with all thalamo-telencephalic and telencephalo-thalamic connections, as well as the prethalamic reticulo-thalamic projection and the subpial optic tract.

The *P2rx7*-EGFP-positive zona limitans line seen at the ependym, named here zona limitans organ (ZLO), shows a continuation that bends caudalwards along the alar-basal boundary. It can be followed back to the thalamo-pretectal limit (ZLO; Figs. 4a, b, 10a); the whole specialization is thus restricted to the p2 prosomere, which contains the Th in its alar plate, and the ZLO limits the Th rostrally and ventrally. It had been already conceived that anteroposterior SHH patterning effects of the zona limitans on the Th might be complemented by analogous ventrodorsal effects from the underlying basal plate (review in (Puelles and Martinez 2013)).

Another embryonically active *P2rx7*-EGFP-positive linear ependymal site appears in the basal Hy, along the linear longitudinal tuberomammillary limit between the perimammillary/periretromammillary (PRM) area and the tuberal/retrotuberal area, forming the *VHO*, where the WNT8b morphogen is selectively produced and released (VHO; Figs. 4b, 10b–d; Garda et al. 2002; Puelles et al. 2012a; Puelles 2017)). This signal is oriented longitudinally, parallel to the hypothalamic floor plate (see cited references), and its location apparently coincides with the locus where a VHO is readily visible in non-mammals (though such an organ is not histologically distinct in mammals; however, see Puelles et al. 2012b; Diniz and Bittencourt 2019). This tuberomammillary linear circumventricular specialization site is also known to be associated to transport of monoamines from blood and cerebrospinal fluid into its ependymal cells and associated proximal and distal neurons. Finally, this locus also expresses the gene coding for histamine hydroxylase, which is associated selectively to the local production of histaminergic neurons (these migrate at short range into tuberomammillary, mammillary or retromammillary (RM) neighborhoods). Recent comparative mappings of melanin-concentrating hypothalamic neurons associate also this population that expands into the overlying mantle to an origin at the VHO locus (irrespective of the name applied to it; (Li et al. 2018); (Diniz and Bittencourt 2019)). An analysis of these properties suggests that an equivalent organ apparently exists as well in mammals, even though it is not easily distinguishable histologically from standard ependym (review in (Puelles et al. 2012b)). This hypothesis is corroborated further by our present observations. The VHO was recently postulated as a novel secondary organizer possibly contributing to the dorsoventral regionalization of the basal Hy into tuberal and mamillary subregions (Puelles 2017).

We also observed another small ependymal specialization that expresses *P2rx7*-EGFP signal intensely. It forms a small ependymal patch located within the tuberal basal hypothalamic region, just intercalated between the distinctly negative ventromedial hypothalamic nucleus (VMH) and the equally negative arcuate nucleus (Arc); we called it the postarcuate organ (PArcO; VMH; Arc; Figs. 4a, 10d–f). This is to our knowledge the first description of this ependymal specialization.

### Other results in the forebrain (telencephalon, hypothalamus, diencephalon and midbrain)

Our mapping stands methodologically on the recently updated and expanded prosomeric model of the forebrain, eschewing the outdated traditional columnar model (Puelles 2013, 2016, 2017, 2019; Puelles et al. 2013, 2012b; Puelles and Rubenstein 2015). The prosomeric model contemplates three primary rostrocaudal forebrain regions (proneuromeres), united under the modern forebrain concept by their sharing of a number of genoarchitectonic features: (1) The secondary prosencephalon (the sum of telencephalon and hypothalamus, including the evaginated neural retina); note the telencephalon is considered within this model as a paired, evaginated, and much overgrown dorsal alar part of the hypothalamus; the mutual relationship between telencephalon and hypothalamus accordingly is dorsoventral. (2) The diencephalon proper (that no longer includes the hypothalamus, and is subdivided segmentally into prethalamus, Th and PT). (3) The midbrain, which ends at the isthmo-mesencephalic boundary (Puelles 2016, 2019; Puelles and Rubenstein 2015). The telencephalon can be further divided conventionally into pallium and subpallium, whereas the hypothalamus, diencephalon and midbrain domains display a general division into alar/roof and basal/floor territories. After covering the forebrain, we will examine the *hindbrain*, which is divided into prepontine, pontine, retropontine and medullary proneuromeric subregions (Puelles 2013; Watson and Puelles 2017), and, finally, will provide some brief comments on the spinal cord.

### Telencephalon

Septum: the most massively *P2rx7*-EGFP-labelled part of the telencephalon was the septum (including both its small pallial and large subpallial parts). This labelling excludes distinctly the vertical and horizontal diagonal band nuclei (VDB and HDB, respectively), as well as their extension into the MS division (VDB, HDB; MS; Figs. 4a–c, 5a, 9c–h). Note that there is a jump between Figs. 9g and h, which, respectively, illustrate levels above and below the ac (not shown), i.e., septal versus preoptic areas. The septal subpallial subregion is regionalized into preoptic, diagonal, pallidal and striatal subdomains (Puelles 2016; Puelles et al. 2013, 2016). Most abundant fluorescent neurons characterized the lateral ventral septal nucleus (LSV) and LSI, as already observed in E14,5 embryos (LSV, LSI; Figs. 2c, d, 4a–c, 5a, b, 9a–f), whereas the labelled population of the LSD was clearly less dense (LSD: Figs. 8f, 9a–d). The TS (TS; Figs. 9a–e), septofimbrial nucleus (SFi; not identified in the Figs.), and septohypothalamic nucleus (SHy; Figs. 4a, b, 9f–h) also contained abundant labelled cells, similarly to the non-septal MnPo (MnPo; Figs. 4c, 9f, g). When favorably sectioned, the SHi appears as a distinct band of strongly labelled elements under the corticoseptal border, which are continuous rostroventrally with the navicular nucleus (Nv), another elongated cell band found just rostral to the nucleus accumbens (Acb) (SHi; Figs. 4a–c, 5a, 8e, f, 9a, b; Nv; Figs. 5a, 8e, f, 9e). There is also a profuse positive septal fiber plexus, which was most dense at the dorsocaudal and ventrocaudal parts of the septum, coinciding with LSI and LSV (see also Fig. 3d).

The commissural septum surrounding the TS and SFi is actually a part of the telencephalic rp (described above separately), and the local positive plexus is excluded from the negative cortically-originated fiber packets of the cc, hc and fx. There is also the intensely positive SFO. One of the parasagittal sections shows a positive IG (IG, i.e., supracommissural hippocampus; Fig. 4b), which is less easily identified in horizontal sections (IG; Figs. 8d–f, 9a, b). We understand the IG as a supracallosal analog of the hippocampal CA3 field, with which it is continuous histologically, as indicated by some molecular markers (Puelles 2019). It is unknown whether the IG has signaling properties. Remarkably, the taenia tecta, widely held to be a precommissural continuation of the IG, and is located rostrally to the SHi and Nv nuclei reaching the medial side of the olfactory peduncle (ped), was wholly unlabeled (not shown).

Cortex: the olfactory bulb (OB) shows a moderately dispersed population of *P2rx7*-EGFP-labelled cells in its inner granular layer (OB; Figs. 4c, 5a, 8a, b), which partly extends within the anterior olfactory nucleus (AON; Fig. 8c, d). Parasagittal sections cutting tangentially the anterior cingulate cortex (ACg) display a well-localized patch of large labelled neurons, lying at some distance from the corticoseptal border. According to rodent atlases, this area possibly corresponds to area 32 (A32), a distinct subregion of the ACg (A32; Fig. 5a). Horizontal sections also reveal this distinct A32 cell population, which may correspond specifically to its layer 5 component (A32; Fig. 8b–d), given its overall continuity with less remarkable cell populations observed in layer 5 of neighboring orbital, frontal (FCx), parietal (PCx), ACg and middle cingulate cortex (MCg) (Lateral orbital cortex (LOCx), Medial orbital cortex (MOCx), FCx, PCx, ACg, MCg; Fig. 8a–f. Note the insular cortex (ICx) only shows dispersed cells in various layers, rather than a compact layer 5 aggregate (Fig. 8c–f). These cortical fields also have some additional labelled neurons in upper layers, as well as in layer 6a, but they are dispersed and low in number. Remarkably, cortical regions placed more caudally show significantly less *P2rx7*-EGFP-positive cells, with a gradual decrease of their number. The relative density of such cortical neurons suggests that they may be interneurons of some type that predominates in layer 5, mixed potentially in A32 and perhaps elsewhere with a particular subgroup of layer 5 pyramids. This latter alternative is partially supported by the observation that labelled axons can be seen streaming out of the labelled layer 5 into the internal capsule (ic; Fig. 7c, d). There is also a labelled local neuropil restricted to isocortical layer 5, which does not extend into the insula (i.e., FCx; PCx; ICx; Figs. 6a–c, 7a–d, 8c–e). This neuropil may belong to local collaterals of efferent pyramidal cell axons, given that thalamic afferents do not target this layer. Separately, there are also *P2rx7*-EGFP-labelled neurons in the hippocampus (Hi) proper. Their selective distribution is best seen in lateral parasagittal sections, which illustrate significant labelling at the CA3 field (not so at CA1, CA2), as well as at the dentate gyrus (DG) (CA3; DG; CA1; Fig. 7c, d). The CA3 cells lie mostly within the alveus and the stratum oriens, which again suggests an interneuronal nature. The DG elements occupy the molecular layer above the negative granule cells (i.e., see DG; Figs. 5b, c, 7c, 9h), thus also implying an interneuronal nature. There are also some dispersed labelled cells at the hilum of the DG, as well as in the neighboring entorhinal cortex (ERh) (Hi; ERh; Fig. 7c, d) and amygdalo-hippocampal area (AHi) (AHi; Fig. 7c, d). In many places of the telencephalon and elsewhere, there is *P2rx7*-EGFP signal along a thin marginal (subpial) lamina. This labelling possibly corresponds to glial endfeet of radial glia cells and/or free astrocytes, where these contact the pial basal membrane; they may become visible as a sheet where they are particularly dense (i.e., lamina marked with an asterisk in Fig. 7a–c).

Subpallium: apart of the subpallial septum, treated above, we observed also *P2rx7*-EGFP signal in the striatal formation, namely a few cells at the olfactory tuberculum (OT) and the Acb (Acb; OT; Figs. 5c, 9d, e). The main mass of the caudo-putamen displays some dispersed labelled neurons, similarly as the strio-amygdaloid area (StA) (St; StA; Figs. 6a–c; 7a–d, 8e, f). These probably represent interneurons according to their apparent density. Separately, another group of labelled cells correlates in position with the substantia innominata and basal nucleus of Meynert (SI/Bas), classified as diagonal domain intermediate stratum components (SI/Bas; Figs. 5c, 9h). The preoptic area is also subpallial, and likewise contains *P2rx7*-EGFP-positive elements. The median locus in front of the ac is occupied by the strongly labelled MnPo (MnPo; Figs. 4c, 9f, g). Next to it, in contact with the fx, lies the SHy, which partly descends into the medial preoptic area (MPo); the latter shows a negative central core, with labelled cells forming a shell around it (SHy; MPo; Figs. 4a, c, 5a, b, 9h, 10a). Many labelled fibers can be seen coursing into the hypothalamus through the lateral shell of the MPo nucleus (MPo; Fig. 5a). The lateral preoptic area (LPo) contains relatively abundant dispersed positive elements (LPo; Figs. 5c, 9h, 10a). There appears also a distinctly labelled superficial population in the anteroventral medial amygdala (MeAV), held to be subpallial (MeAV; Fig. 6a, b).

### Hypothalamus

There is only limited cellular labelling in the hypothalamus, though most of the alar and basal hypothalamus field shows some background neuropil signal, probably resulting from afferent fibers coming from the septal and preoptic areas. This background neuropil contrasts with some hypothalamic nuclei which stand out as lacking completely such labelled input. One clear-cut example of absolute lack of signal is the large VMH, found in the basal tuberal area (VMH; Figs. 4a, b, 5a, 10c–f). The medial mamillary body (MM) has similar characteristics (MM; Fig. 5a). There are nevertheless two distinctly *P2rx7*-EGFP-positive cell populations in the alar hypothalamus, both in a relatively rostral locus. The supraoptic nucleus (SpO) appears subpially within the paraventricular area, opposed to the dorsal aspect of the optic chiasma and the optic tract, and consists of a longitudinal population of strongly positive neurons (SpO; Fig. 10a). This subpial cell group defines the thin dorsalmost part of the alar hypothalamus, which is located above the optic tract, and is a landmark for the telencephalo- or preopto-hypothalamic border. The labelled SpO cells follow the longitudinal optic tract exactly up to the place where it starts to course over the cerebral peduncle; this corresponds to the boundary between terminal and peduncular parts of the hypothalamus, derived, respectively, from hypothalamic prosomeres 2 and 1 (hp2 and hp1, respectively) (Puelles et al. 2012b; Puelles and Rubenstein 2015). More ventrally in the alar hypothalamus, alevel with the subparaventricular area, we see at both sides of the acroterminal rostral end of the hypothalamus intense *P2rx7*-EGFP signal at the suprachiasmatic nucleus (SCh); some images suggest a positive capsular subregion surrounding a negative core part (SCh; Figs. 4a, b, 10b–d).

The alar-basal boundary separates the SCh nucleus from the basal tuberal area, occupied mainly by the negative VMH nucleus and the acroterminal (rostromedian), similarly negative Arc. As mentioned above, here, roughly at the ventricular border between VMH and Arc, is where we distinguish at the local ependym by its strong *P2rx7*-EGFP signal what seems a novel circumventricular organ, named by us PArcO (PArcO; Figs. 4a, 10d–f; note there are right and left organs, practically in contact, due to the narrowness of the local ventricle). Topologically ventral to the tuberal basal subregion of the hypothalamus is the MM-RM basal subregion (MM; RM; Fig. 4a–b). Along the mutual tuberomamillary boundary, there extends a thin intermediate basal band where the histaminergic neurons of the tuberomamillary hypothalamus are produced (Puelles et al. 2012b). The ependym of this thin tuberomamillary or intermediate basal strip displays circumventricular organ histological characteristics in non-mammalian vertebrates, defining the so-called ‘VHO’, a topologically longitudinal ependymal specialization we mentioned above. This ependymal organ is less distinctive (cryptic) in mammals, but (Puelles et al. 2012b) argued that, though modified in certain respects, a VHO homolog may exist in mammals, judging on the basis of various conserved molecular features (see also Diniz and Bittencourt 2019). Importantly, this site has been suggested to represent in embryos a secondary organizer where the WNT8B morphogen is released into the surroundings (i.e., probably affecting dorsoventral patterning of the hypothalamic tuberal versus mamillary territories; see (Puelles 2017). Our present material shows a distinct line of *P2rx7*-EGFP-positive VHO ependymal cell bodies (with weaker signal than the PArcO) at the locus of the tuberomamillary or intermediate basal hypothalamic stripe (VHO; Figs. 4b, 10b–d).

### Diencephalon

We already mentioned *P2rx7*-EGFP expression at the non-chorioidal diencephalic rp mentioned above, as well as at the ependymal ZLO, which delimits rostrally and ventrally the Th. The diencephalic region otherwise displays *P2rx7*-EGFP-positive cells only within the Th and the PT.

At the Th, we identified medially positive cells at the medio-dorsal nucleus (MD; Fig. 5a–c). The periventricular and posterior thalamic complexes seem devoid of signal. More laterally, we detect again labelled cells at the ventrobasal complex (VB) and the superficial lateral dorsal nucleus (LD) (VB; LD; Fig. 6a, b). Labelled fibers apparently descending from isocortical layer 5 via the ic bend into the prethalamus and Th across the Rt (ic; Rt; Th; Figs. 1, 6a, b). They cross longitudinally the Rt region (otherwise unlabelled) and penetrate the VB (Fig. 6a). Some of these fibers seem to course longitudinally across Th and PT, maybe reaching the midbrain tectum.

The PT is divided anteroposteriorly into three transverse alar domains, identified according to their relationships with the pc (precommissural (PcPT), juxtacommissural, commissural (CoPT) PT sectors; (Ferran et al. 2008)). Labelled cells are found mainly at the PcPT and CoPT sectors (PcPT, CoPT; Figs. 5b, 6a, b, 9f, g). The largest nucleus in the PcPT is the anterior pretectal nucleus (APT), whereas the parvocellular and magnocellular nuclei of the pc (PcPC and McPC, respectively) are distinct commissural nuclei (i.e., APT; PcPC; McPC; Fig. 5c, and APT in Fig. 9f). The diencephalo-mesencephalic boundary lies just behind the commissural PT (and the pc).

### Midbrain

The alar mesencephalon consists of a rostrocaudal series of four different structural entities: the TG, the SC, the IC and the preisthmus (PIsth). The literature usually mentions exclusively the SC (lumping TG into it) and IC (lumping the PIsth therein), but the four domains are distinct both molecularly and anatomically (Puelles et al. 2012a; Puelles 2016). The TG was classically mislabeled as ‘posterior pretectal nucleus’, and the PIsth roughly corresponds to the classic ‘cuneiform area’ (see also (Puelles 2019)) (TG; SC; IC; PIsth; Figs. 4–7). *P2rx7*-EGFP-positive cells are mainly present in the SC, characterizing particularly its periventricular sector within the periaqueductal gray (PAG) (SC; PAG; Figs. 4a–c, 5b, c, 9f–h), but there is also a distinct labelled population in SC´s superficial stratum (SS) (SC; SS; Figs. 6a, b, 9f, g). In the caudal half or so of the midbrain, the characteristic balloon-shaped neurons of the mesencephalic trigeminal nucleus (Mes5) also appear labeled just outside the PAG (Mes5; Figs. 6a, 10c–f). In contrast with the SC, its rostral and caudal neighbors, the TG and IC formations, are basically negative for our marker (TG; IC; Fig. 6a–c), with possible exception of a cell stratum found deep to the main central nucleus of the IC (Fig. 9h). In lateral sagittal sections, we can see an oblique packet of positive fibers separating the SC from the IC (Fig. 6b, c), which converges medially into the stratum album centrale (SAC) (tectal white matter; Fig. 6a), and through it reaches the rp and the icc and tc (icc; tc; Figs. 5b, 6b). The laterally coursing fiber packets thus represent the labelled axons of commissural neurons of the SC and IC (but not of TG or PIsth). The PIsth formation only shows positive cells laterally, where we see the cuneiform nucleus (Cnf) at intermediate level (Cnf; Fig. 6a), and the elongated subbrachial nucleus (SubB) superficially (SubB; Fig. 6b, c).

### Hindbrain

As stated above, within the prosomeric model, the hindbrain is divided rostrocaudally into prepontine, pontine, retropontine and medullary proneuromeres, each of them subdivided into rhombomeres (r) to a total of 12 such units ordered rostrocaudally (r0-r11; r0 corresponds to the Isth; review in Puelles 2013; Puelles et al. 2013; Watson and Puelles 2017; Watson et al. 2017).

Prepontine level: this is the portion of hindbrain that lies rostral to the pontine bulge and reaches the isthmo-mesencephalic boundary (Puelles 2016, 2019). It contains the massive Cb dorsally plus the derivatives of the Isth –r0- and r1; in older times this prepontine region was wrongly included in the midbrain, and more recently, it was also often wrongly held that the Isth was a rostral part of r1. Recent *Fgf8*-based transgenic fate-mapping performed by (Watson and Puelles 2017; Watson et al. 2017) demonstrated the full independence of the Isth, or r0, from r1. Both prepontine rs contribute to distinct parts of the Cb (vermis in r0 versus hemispheres in r1), the whole isthmo-cerebellar complex being embryologically independent from the more caudal pons (r2-r4), as opposed to classic assumptions. The nuclear derivatives of this rostralmost hindbrain region are discussed in (Puelles 2016, 2019).

At medial isthmic levels, we detected *P2rx7*-EGFP-positive neurons within the dorsal raphe nucleus (DR) and the rostral part of the interpeduncular nucleus (IP) (Isth; DR; IP; Figs. 4a–c, 5a–c, 10e, f). More laterally (alar plate), we observe the isthmic and labelled parabigeminal nucleus (PBG; a population interconnected with the SC) and the microcellular tegmental nucleus (MiTg) (PBG; MiTg; Fig. 6a, b). As regards r1, *P2rx7*-EGFP-positive neurons in the caudal non-isthmic part of the DR (DR; Fig. 4a, b), as well as in central gray (CG) populations surrounding the negative dorsal tegmental nucleus (DTg), including the spheroid nucleus (Sph) and the laterodorsal tegmental nucleus (LDTg) (DTg; Sph; CG; Figs. 4b, 5a, b, LDTg; Figs. 10e, f, 11a–c). These formations are developmentally (and functionally) related to the interpeduncular complex (Lorente-Canovas et al. 2012). More laterally, r1 shows positive neurons at the dorsal nucleus of the lateral lemniscus (DLL; Fig. 6c), as well as in a population corresponding to caudally migrated cells of the Mes5 (Figs. 6a, 11b, c).

The Cb, as said, derives its vermis from the Isth and its hemispheres from right and left r1. Labelled cells mark distinctly at E18.5 the still immature Purkinje cell layer (Pk), as well as the EGL, where cerebellar granule cells are produced (Pk; EGL; Figs. 5a–c, 6a–c). The EGL already appeared labeled at E14,5 (Fig. 2b).

Pontine level: this hindbrain portion comprises r2-r4, which enclose ventrally the migrated basilar pontine nuclei (Pn; originated at the rhombic lip (RhL) at r6-r8 levels), as well as the lateral root of the trigeminus. The Pn proper aggregate only within r3 and r4. However, the cerebellopetal axons of this population (forming the middle cerebellar peduncle) are not confined in their course to r3-r4, and spread out also through r2 (surrounding there the trigeminal root) to access the Cb through r1 (the closest entrance). The pontine population expresses our marker strongly, which is present both in the postmigratory basilar Pn and in the subpial elements of the corpus pontobulbare (CPB), still migrating at E18.5 (Figs. 4a–c, 5a–c, 6a, 11b–d). Other positive neuronal formations found in the pontine region include medial cells of the reticulotegmental nucleus (RtTg) (another migrated rhombic-lip-derived component of the pontine complex) and the pontine raphe nucleus (PnR) (RtTg; PnR; Fig. 4a, b), as well as radially distributed cells of the intermediate reticular area (IRt) at CG, intermediate and superficial radial levels (IRt; Fig. 11d, e). We also see positive cells within the corresponding portion across r2-r4 of the vestibular sensory column, in particular the superior, medial and lateral vestibular nuclei (SVe, MVe, and LVe, respectively) (SVe; MVe; LVe; Figs. 5b, c, 6a–c, 7a, b, 11e).

Retropontine level: this hindbrain sector encompasses only r5 and r6. In the past, it used to be wrongly included in the pons, because the particularly disproportionate growth of the r3-r4 basilar Pn in the human brain caused them to overhang the ventral aspect of the neighboring r5–r6 units (forming the *foramen caecus*). This gave the false impression that the latter’s contents were located within the pons; actually, such overlap is not present in the brain of small mammals, including the mouse. The r5 unit is the one that contains characteristically the abducens motor nucleus and nerve root, and the visceromotor facial nerve efferent component (superior salivatory nucleus). Likewise, it also contains the superior olivary complex and associated trapezoid body (Tz) decussation of the ascending lateral lemniscus auditory pathway. The latter crosses the ventral midline in ventral r5, caudal to the pons proper (Tz; Figs. 4a–c; non-labeled). The r6 unit is the one that receives the postmigratory branchiomotor facial motor (7 N) nucleus born in r4 (non-labelled; see 7 N in Fig. 5c), and likewise contains the glossopharyngeal nerve (9 N) root (9 N; Fig. 6b). In our material, the only derivatives of r5 and r6 that express *P2rx7*-EGFP are the corresponding segmental units of the vestibular and cochlear sensory columns (see Figs. 5, 6, 7), as well as the caudal continuation of the radial IRt pattern described in the pontine region (IRt; Fig. 12a–c).

Medulla oblongata level: this classic caudal portion of the hindbrain tends to have a repetitive segmental structural pattern, which led classic anatomists to treat it as an undivided unit. However, appropriate gene markers have recently divided it into cryptic r7-r11 (Tomas-Roca et al. 2016); these are not well delimited anatomically but are distinct molecularly. The cranial nerve roots associated to this sector are the vagus (10 N; Fig. 6b), accessory or spinal (11 N), and hypoglossus (12 N) nerves. Both the trigeminal descending sensory column (DS5) and the viscerosensory column (solitary tract (Sol) nucleus) are conspicuously absolutely negative for our marker (DS5; Figs. 6b, c, 7a, 12a–g; Sol; Figs. 5b, c, 12c–g). The Sol complex appears limited laterally by a shell of positive fibers, which separate it from the likewise negative dorsal column nuclei, the gracilis and cuneatus nuclei (Gr and Cu, respectively). At the rostral end of this latter complex, there appears superficially the external cuneate nucleus (Ecu), which displays a cerebellopetal population of positive neurons (ECu; Figs.5c, 6a, b, 12b). We also see labelled at these medullary levels the IRt, implemented as a radial complex at periventricular, intermediate and even superficial levels, possibly including the correlative packets of radial glia, and leaving unlabelled the adjacent medial and lateral reticular formation components (IRt; Fig. 12c–g).

Spinal cord level: At the spinal cord the DS5 is substituted by the dorsal horn (DH), which is likewise negative (DH; Fig. 12h, i), the Sol column reaches its end and the IRt formation is substituted by the so-called spinal intermediate zone (IZ), with similar staining characteristics (IZ; Fig. 12h, i). Superficially, the ventral horn (VH) appears, with a number of positive cells, probably of interneuronal nature (VH; Fig. 12h, i).

## Discussion

### Molecular aspects

P2X7R stands out within the purinergic receptors due to its particular properties. Compared to all other P2X receptor subtypes, P2X7R has distinct structural and functional features (Surprenant et al. 1996). Unlike other P2X subunits, P2X7R has a significantly longer C-terminus consisting of 293 amino acids and does not seem to heterotrimerize with other members of the P2X family. The C-terminus of the P2X7R has been implicated in regulating receptor function, including signalling pathway activation, cellular localization, protein–protein interactions, and post-translational modification (Costa-Junior et al. 2011). Finally, P2X7R has an affinity for ATP much lower than other P2X receptors (EC_50_ ≥ 100 μM), and its prolonged activation does not result in desensitization of the receptor (Surprenant et al. 1996). Moreover, a growing number of brain physiological functions are associated to the activity of this receptor (Huang et al. 2019; Jimenez-Mateos et al. 2019; Kanellopoulos and Delarasse 2019; Miras-Portugal et al. 2016, 2019b; Oliveira et al. 2016), highlight the relevance of comprehensive analysis of the expression pattern of the P2X7R that has been performed in this study.

### The septum and circumventricular organs

Notably, the strongest *P2rx7*-EGFP signal was found in the septum. Previous studies also showed an important presence of P2X7R mRNA in this area within the adult rat brain (Yu et al. 2008). This area has been traditionally associated to reward and reinforcement functions (Olds and Milner 1954), which goes in line with recent studies showing the involvement of the P2X7R on reward enhancement produced by psycho-stimulant drugs (Gentile et al. 2019). Likewise, a remarkable high level of *P2rx7*-EGFP expression was detected in the SFO, a circumventricular organ of the brain associated to osmoregulation and fluid balance (McKinley et al. 2019). Interestingly, P2X7R has been proposed as an attractive target to control blood pressure in chronic kidney disease, since its antagonism would increase renal medullary perfusion, reduce interstitial inflammation, prevent interstitial cell death, and improve blood pressure control (Menzies et al. 2015). Moreover, *P2rx7*-EGFP signal was also prominent at other circumventricular organs or specializations. We provide next some background on the ependym and circumventricular organs.

The normal ependym that forms at the apical or ventricular surface of the neural wall (lining the ventricular cavities) is the direct derivative of the early embryonic neuroepithelium. What was interpreted by pioneering neurohistologists as a distinct monocellular layer of small cuboidal epithelial cells (known as ependymocytes) contains indeed the cell bodies of neuroepithelial ependymal cells, also known alternatively as radial glial cells, forming an epithelial sheet interconnected by cell–cell adhesions. However, the ependymocytes are not strictly cuboidal in shape, since they also extend a radially oriented basal cytoplasmic process reaching the outer or pial surface of the brain wall (Donkelaar 2020; Nieuwenhuys 2016). They resemble in this the immature neuroepithelial cells, whose somata also concentrate next to the ventricular surface, building the pluristratified embryonic ventricular zone, where mitotic activity is mainly observed and young neurons are born. As development advances and proliferation/neurogenesis slows down, the remnant of the ventricular zone normally transforms into the thinner, apparently monostratified ependym. The ependym keeps a virtual mitotic potency, normally repressed by lateral inhibition.

In addition, at some precise brain locations, there develop specializations of the ependym (usually thickenings), which are called circumventricular organs. These are thought to perform a particular sensory or secretory function (Ganong 1977; Johnson and Gross 1993; Vigh et al. 2004; Duvernoy and Risold 2007; Kawano and Masuko 2010; Kiecker 2018). A typical secretory ependymal specialization is the pretectal *SCO*, a large ependymal thickening found under the pc in the pretectal roof region (its cells release a mucoid secretion —known as Reissner’s fibre— into the ventricular cerebrospinal fluid, whose function remains unclear; (Grondona et al. 2012; Muñoz et al. 2019)). In other circumventricular organs, there appear one or more sorts of neurons associated to the ependymal thickening; these neurons may be attached to the ventricular lining (i.e., sending a dendrite into the ventricular fluid), or may be free in the neighborhood. Sometimes they form distinct layers (known as proximal and distal cells); there may exist neuropil layers as well, in a corticoid arrangement (Vigh-Teichmann and Vigh 1983). The complex may show specific afferences and efferences, indicating its participation in a particular signaling circuit (e.g., (Iwanaga et al. 2019)). A typical example of such complex circumventricular organs is the *VHO*, located longitudinally in the basal hypothalamus (Puelles 2017). Its ependymal cells are known to recapture and/or synthetize monoamines (see (Xavier et al. 2017)) and have also been related to the production of hypothalamic histaminergic cells and melanin-concentrating hormone neurons, which tend to migrate to more superficial levels of the mantle zone in various vertebrate lineages (Puelles et al. 2012a, b; Xavier et al. 2017; Diniz and Bittencourt 2019). The median SFO, found under the ventral hc (a telencephalic rp locus), is singularly both circumventricular and subpial, analogously as the hindbrain *AP* (Fig. 1).

Combined ependymo-neuronal organs apparently detect and measure a given biological signal, representing a sensory node from where changes in the measured variable trigger reflex neuronal reactions leading presumably to homeostasis (Vigh et al. 2004). However, we still do not know well enough the functions of many of those circumventricular specializations. Interestingly, at embryonic stages, some of such specializations may act as secondary organizers that release morphogens that diffuse into neighboring neural territories, establishing concentration gradients that function as positional information used for regionalization (Puelles et al. 2012b; Puelles 2017). Secondary organizers express genes coding for secreted morphogens, whose gradients aid differentiation of the surrounding field into smaller areal domains, depending on the morphogen concentration. Neighboring or distant neuroepithelial progenitor cells capable of detecting given levels of these signals as positional information (i.e., distance from the signal source or sink) may select a particular genomic pathway for the specification of their neuronal derivatives (Wolpert 1969, 1971).

Along this line, P2X7R has been shown to modulate neural ventricular progenitors as well as their progeny. For instance, this receptor contributes to maintain ESC proliferation, whereas its inhibition results in the promotion of differentiation (Glaser et al. 2014). Moreover, P2X7R is also expressed in embryonic telencephalon neurosphere cultures, and the downregulation of this receptor induces an increase in differentiation (Oliveira et al. 2015). Regarding neural cell populations, the activation of P2X7R in astrocytes inhibits fibroblast growth factor 2-induced proliferation (Neary et al. 2008) and promotes the differentiation of neuroblastoma N2a cells via the regulation of the Ca^2+^⁄calmodulin-dependent kinase II signalling cascade (Gomez-Villafuertes et al. 2009). In addition, the strong *P2rx7*-EGFP signal displayed in some ependymal sites, including the hypothalamic *VHO* and *PArcO* organs, also suggest a potential role of P2X7R in the long-distance volume transmission by neuropeptides due to innervation of the ependymal cells and associated neurons, as has been recently shown, precisely, in the hypothalamus (Alpar et al. 2019).

Another potential embryonic function of such signaling ependymal specializations and their basal processes might be involved in guiding (attracting or repelling) axonal navigation, or, eventually, tangential neuronal migration (Puelles 2017). This fact is especially interesting since *P2rx7*-EGFP signal is detected in several neuronal populations and also in selected packets of fibers and terminal neuropils. These patterns are differentially specified, since they appear next to neural fields entirely lacking such fibers, as found at most brain regions. Precisely, many purinergic receptors and their downstream signalling proteins control adhesion and migration, a cyclic process that involves cell polarization, membrane protrusion at the leading edge, focal adhesions, stress fibre formation, cell contraction, and retraction of the trailing edge to allow cells to move forward. For instance, the neuronal surface glycoprotein, Thy-1, binds to astrocytes and thereby promotes adhesion and migration by interaction with the αVβ3 integrin and syndecan-4 receptors. Interestingly, these interactions activate a signalling cascade that release of ATP to the extracellular milieu, where it activates P2X7R (Alvarez et al. 2016; Lagos-Cabre et al. 2017). P2X7R also act as negative modulator of neurite extension. Thus, its expression is decreased in retinoic acid-differentiated neuroblastoma N2a cells (Wu et al. 2009). Moreover, either pharmacological inhibition or downregulation of P2X7R induces neurite outgrowth of N2a cells (Gomez-Villafuertes et al. 2009). In agreement with this finding, an increase in intracellular Ca^2+^ levels in axonal growth cones mediated by P2X7R activation reduces axonal elongation in cultured hippocampal neurons (Diaz-Hernandez et al. 2008). In the same cellular model, cleavage of extracellular ATP by tissue-nonspecific alkaline phosphatase prevents P2X7R activation, thus promoting axonal growth (Diez-Zaera et al. 2011). Therefore, P2X7R activity might be involved in the guidance of developing axons to their correct destination.

### The neural roof plate and related commissures

The presently discovered strong topographic association of *P2rx7*-EGFP signal to the radial astroglial palisades constituting the neural (non-chorioidal) rp of the brain, is so far unique (not shown by any other molecular marker studied), and suggests a role in guiding axons into the formation of a variety of brain dorsal commissures. Genes coding for dorsalizing morphogens of the Wnt and BMP families at the embryonic rp may lie upstream of the activation of P2X7R at this site.

The neural rp derives from the early union between the right and left ridges limiting primarily the neural plate during the process of neurulation, i.e., closure of the neural tube. The resulting fused median rp begins rostrally at the ac level (experimental analysis by Cobos et al. 2001; Puelles et al. 1987); see also (Puelles 2018)), and extends down to the caudal end of the spinal cord. This median embryonic locus becomes a source of dorsalizing morphogens, which diffuse ventralwards into the lateral wall of the neural tube, antagonizing the ventralizing morphogens that diffuse in opposed direction from the notochord-induced floor plate. Antagonic roof and floor signals thus jointly orchestrate a crucial dorsoventral patterning mechanism affecting the differentiation of roof, floor, alar and basal subdivisions of the adult brain (review in Puelles 2017).

In the course of development, two separate rp patches, one in the forebrain (bridging rostral Th, prethalamus and caudal telencephalon) and another in the hindbrain (bridging the distance between the Cb and the obex), differentiate into a flattened sort of neuroepithelium, the so-called ch (Puelles 2013). The later adheres to invaginating blood capillaries and participates in the formation of intraventricular chorioidal plexi. This is where the cerebrospinal fluid that fills the brain ventricles and floats the brain in its bone case is filtered out of blood plasma. These highly vascular chorioidal roof sites do not participate at all in the mapping of *P2rx7*-EGFP that occupies us here. The rest of the rp appears in three parts that acquire neural structure and are separated by the ch [(1) the telencephalic Scorp, including all telencephalic commissures, (2) the diencephalo-mesencephalic and isthmo-cbrp incorporating habenular, posterior, tectal intercollicular and cerebellar commissures, and (3) the obex at the caudal medulla and the spinal rp]. Depending on the location, the neurally differentiated rp may produce a variety of neurons and specialized median radial astroglia cells, or, else, differentiate as a circumventricular sensory organ (i.e., SFO) or a glandular ependymal specialization (SCO under the pc; Fig. 1) (Puelles 2013).

Neuroanatomy textbooks do not dedicate much space to the detailed histological structure of the non-chorioidal parts of the brain rp. Leaving occasional neurons aside, in many places, a simple (median) or double longitudinal palisade of astrocytic radial glia cells differentiates. These median glial cells relate intimately with packets of axons which cross the rp, forming roof commissures or decussations. In embryos, these roof glial cells may attract and guide mechanically and chemically the axons, so that they grow efficiently across the rp. Little or no neurogenesis characterizes the ‘commissural’ roof sites. This is the case in the telencephalic septocommissural region (ac, hc and cc commissure), the caudal diencephalic roof surrounding the pineal gland (pi) (habenular commissure) and across the pretectal roof (pc). The midbrain tc and icc are also associated to poor neurogenesis. The isthmus contains the trochlear nerve decussation, and participates with r1 in the cryptic cerebellar commissure (hidden in the cerebellar white matter). If we jump over the hindbrain chorioidal patch to the medullary obex roof, we find there the commissure of the Sols (commissura infima). Even the spinal cord displays a dispersed dorsal commissural system of propiospinal fibers, which cross the glial spinal roof palisade (Puelles 2013).

At some restricted loci, the non-chorioidal rp neuroepithelium differentiates (often under the local commissure) to form singular ependymal specializations identified as either circumventricular organs or secretory organs (Diniz and Bittencourt 2019; Duvernoy and Risold 2007; Ganong 1977; Johnson and Gross 1993; Kawano and Masuko 2010; Kiecker 2018; Vigh-Teichmann and Vigh 1983; Vigh et al. 2004; Xavier et al. 2017). The former receptor-like specializations are able to sense one or more molecular variables either in blood plasma or in the ventricular fluid, and are able to transform such readings, triggering efficient reflex neuronal and humoral signaling that serves homeostasis of the relevant variable. For instance, at the septal roof, behind the ventral part of the hc, we find a sensory organ mediating hydric homeostasis, the *circumventricular* SFO (SFO; Fig. 1). There is also the glandular *SCO* (SCO; Fig. 1) at the pretectal roof, under the pc (it secretes into the ventricle a mucoid substance with as yet unknown function). The *AP* at the medullary obex roof may be regarded likewise as a vagal reflex-mediating circumventricular sensory roof organ, which reacts to toxic molecules in the blood, triggering vomit and other visceral reflexes (Fig. 1).

### Other patterns

The significant labelling observed in several packets of fibres, interneuron populations (for instance in layer 5 of cortex) as well as in radial glia further supports the presence of P2X7R in neuronal and glial populations. Indeed, expression of P2X7R has been found in neurons, astrocytes and oligodendrocytes (Carrasquero et al. 2009; Metzger et al. 2017; Sperlagh and Illes 2014). However, a recent debate has questioned the expression of functional P2X7R in neurons (Illes et al. 2017; Miras-Portugal et al. 2017). Certainly, it has been shown that glial cells might be responsible for the P2X7R-mediated effects firstly attributed to hippocampal neurons (Khan et al. 2018; Rubini et al. 2014). However, the characteristic response following stimulation of this receptor has been demonstrated in cultured neurons or synaptosomes by electrophysiological recordings, intracellular Ca^2+^ measurements, and activation of intracellular signalling cascades (Anderson and Nedergaard 2006; Deuchars et al. 2001; Diaz-Hernandez et al. 2008; Hervas et al. 2003; Ortega et al. 2009). Thus, the results observed in the present study represent an additional value to the line of research supporting the specific presence of the P2X7R in selected groups of neurons.

The labelling found in other specific areas, described subsequently, results also of interest considering certain functions associated to this receptor. We found positive *P2rx7*-EGFP signal on the hypothalamic SCh, which functions as the master circadian pacemaker (Golombek and Rosenstein 2010; Moore 2007). Furthermore, several studies reported GSK3 as a key protein in the regulation of the circadian rhythms controlled at this area (Besing et al. 2015; Costemale-Lacoste et al. 2018; Paul et al. 2016). Interestingly, we have previously described a precise control of GSK3 activity exerted by P2X7R-triggered signalling in cerebellar granule neurons (Ortega et al. 2009, 2010), which suggests a potential role of this receptor in the modulation of daily rhythms. Another interesting area that displays a significant labelling is the hindbrain. The DR, the developing Cb and the Ecu stand out for their known relationship with P2X7R activity. P2X7R has been shown to mediate the release of serotonin in the Hi through their activity in the raphe nucleus (Goloncser et al. 2017). Regarding the Cb, we observed a strong *P2rx7*-EGFP signal located in the EGL, known to produce granule neurons destined to reside in the internal granular layer (Alvarez Otero et al. 1993; Hallonet et al. 1990). Along this line, we have previously described a crucial role of signalling pathways triggered by P2X7R in neuroprotection against trophic factor withdrawal and glutamate-induced excitotoxicity in cerebellar granule neurons (Ortega et al. 2009, 2010, 2011; Queipo et al. 2017). Considering the positive Ecu, a pre-cerebellar cell population jointly with the likewise positive Pn, reticulotegmental nucleus and mesV population, co-localization of P2X7R and glutamatergic synaptic terminals in this area has been previously reported in the rat CNS (Atkinson et al. 2004). Finally, at the spinal cord level, we found a strong *P2rx7*-EGFP staining in the VH, where the expression of P2X7R in synaptic terminals has also been observed in rat CNS (Deng and Fyffe 2004).

In summary, we have described in the present manuscript a notable and specific labelling of the P2X7R in multiple areas of the mouse embryonic brain. Moreover, a circumventricular ependymal specialization previously postulated by (Puelles et al. 2012) to exist not only in non-mammals, but also in mammals, the *VHO*, has been further corroborated by our mouse data. In addition, as a consequence of our analysis, we also described a new hypothalamic ependymal organ (*PArcO*) with unknown associated functions yet to be elucidated. Altogether these results further support the presence of P2X7R on neuronal populations and their axonal processes down to terminal synaptic fields, apart its presence in specialized radial astroglia and some circumventricular organs. This underlines the important role that the purinergic system, and specifically this receptor, may exert in the development and various functions of the mammalian CNS.

## Supplementary Information

Below is the link to the electronic supplementary material.

Supplementary Fig.1 Distribution pattern of EGFP reporter and P2X7R in brain sections from P2rx7-EGFP mouse embryos. a Co-labeling of brain slice from E14 embryo with anti-GFP antibody (green), anti-P2X7 receptor antibody (red) and DAPI (blue). Representative results from n = 3 mice are shown. Scale bar: 50 μM, b Orthogonal projection analysis of a magnified subfield from merge image shows the high co-localization of EGFP signal and P2X7R expression (PDF 5257 KB)

## Data Availability

All the data and material employed in this manuscript is available for revision.

## Abbreviations

[5] or 5: Cortical layer 5
5n: Trigeminal nerve
7N: Branchiomotor facial nerve
8n: Vestibulocochlear nerve
9N: Glossopharyngeal nerve
10N: Vagus nerve
11N: Accessory or spinal nerve
12N: Hypoglossus nerve
3v: 3Rd ventricle
4v: 4Th ventricle
A: Amygdala
ac: Anterior commissure
Acb: Nucleus accumbens
ACg: Anterior cingulate cortex
AHi: Amygdalo-hippocampal area
Am: Medial amygdala
AON: Anterior olfactory nucleus
AP: Area postrema
aprp: Area postrema roof plate
APT: Anterior pretectal nucleus
Aq: Aqueduct
Arc: Arcuate nucleus
AT: Acroterminal domain
A32: Area 32
bas ep: Basal ependym
Calb1: Calbindin
Cb: Cerebellum
CbN: Cerebellar nuclei primordia
cbrp: Cerebellar roof plate
cc: Corpus callosum
CG: Central gray
ch: Chorioidal tela
CM: Centromedial thalamic nucleus
Cnf: Cuneiform nucleus
CNS: Central nervous system
CoPT: Commissural pretectal
CPB: Corpus pontobulbare
Cu: Cuneatus nucleus
Cx: Cortex
DCo: Dorsal cochlear nucleus
DG: Dentate gyrus
DH: Dorsal horn
DLL: Dorsal lateral lemniscus
DMH: Dorsomedial hypothalamic nucleus
DR: Dorsal raphe nucleus
DS5: Descending sensory column
DTg: Dorsal tegmental nucleus
DVe: Descending vestibular nucleus
Ecu: External cuneate nucleus
EGL: External granular cell layer
ERh: Entorhinal cortex
ESC: Embryonic stem cell
FCx: Frontal cortex
fi: Fimbria
fx: Fornix tract
Gr: Gracilis nucleus
GSK3: Glycogen synthase kinase 3
HB: Habenula
hc: Hippocampal commissure
HDB: Horizontal diagonal band nucleus
HDG: Hippocampal dentate gyrus
Hi: Hippocampus
hp1: Hypothalamic prosomere 1
hp2: Hypothalamic prosomere 2
HVO: Hypothalamic ventricular organ
Hy: Hypothalamus
IC: Inferior colliculus
ic: Internal capsule
icc: Intercollicular commissure
ICo: Intercollicular commissure
ICx: Insular cortex
IG: Indusium griseum
IO: Inferior olive
IP: Interpeduncular nucleus
irp: Isthmic roof plate
IRt: Intermediate reticular area
Isth: Isthmus
IZ: Intermediate zone
LC: Locus coeruleus
LD: Lateral dorsal nucleus
LDTg: Laterodorsal tegmental nucleus
LH: Lateral hypothalamus
ll: Lateral lemniscus tract
LL: Nucleus of lateral lemniscus
LOCx: Lateral orbital cortex
LPo: Lateral preoptic area
LSD: Lateral dorsal septal nucleus
LSe: Lateral septal mantle
LSI: Lateral intermediate septal nucleus
LSV: Lateral ventral septal nucleus
lt: Lamina terminalis
LVe: Lateral vestibular nucleus
MB: Midbrain
MCg: Middle cingulate cortex
McPC: Magnocellular posterior commissure nucleus
MD: Medio-dorsal nucleus
MeAV: Anteroventral medial amygdala
Med: Medulla oblongata
Mes5: Mesencephalic trigeminal nucleus
merp: Medullary roof plate
mfb: Medial forebrain bundle
mfp: Mesencephalic floor plate
MiTG: Microcellular tegmental nucleus
MM: Medial mammillary body
MnPo: Median preoptic nucleus
MOCx: Medial orbital cortex
MPo: Medial preoptic area
mrp: Midbrain roof plate
MS: Medial septal
MVe: Medial vestibular nucleus
Nv: Navicular nucleus
OB: Olfactory bulb
OT: Olfactory tuberculum
PAG: Periaqueductal gray
PAGD: Dorsal part of periaqueductal gray
Pal: Pallidum
PArcO: Postarcuate organ
PBG: Parabigeminal nucleus
pc: Posterior commissure (fibers)
PcPC: Parvocellular posterior commissure nucleus
PcPT: Precommissural pretectal
PCx: Parietal cortex
PDTg: Posterodorsal tegmental nucleus
ped: Peduncle
PHy: Peduncular hypothalamus
pi: Pineal gland
Pir: Piriform cortex
PIsth: Preisthmus
Pk: Purkinje cell layer
Pn: Pontine nuclei
Pn migr: Pontine migration or corpus pontobulbare CPB)
PnR: Pontine raphe nucleus
POA: Preoptic area
poc: Posterior commissure
Pr: Prepositus hypoglossi nucleus
PreP: Prepontine hindbrain
PRM: Periretromammillary area
PS5: Principal sensory trigeminal nucleus
PT: Pretectum
PTh: Prethalamus
P2 receptors: Purinoceptors
P2X7R: P2X7 receptor
r: Rhombomere
ReP: Retropontine hindbrain
rf: Retroflex tract
RhL: Rhombic lip
RM: Retromammillary body
rp: Roof plate
Rt: Reticular nucleus
RtM: Medial reticular formation
RtTg: Reticulotegmental nucleus
SAC: Stratum album centrale of superior colliculus
SC: Superior colliculus
scbp: Spinal cord basal plate
SCh: Suprachiasmatic nucleus
SchO: Suprachiasmatic organ
SCO: Subcommissural organ
Scorp: Septocommissural roof plate
SHi: Septohippocampal nucleus
SHy: Septohypothalamic nucleus
SI: Substantia innominata
SI/Bas: Substantia innominata/basal nucleus of Meynert
SFi: Septofimbrial nucleus
SFO: Subfornical organ
SNC: Substantia nigra pars compacta
Sol: Solitary tract
Sph: Spheroid nucleus
SpO: Supraoptic nucleus
SS: Superficial stratum of superior colliculus
St: Striatum
StA: Strio-amygdaloid area
SubB: Subbrachial nucleus
SVe: Superior vestibular nucleus
tc: Tectal commissure
Tel: Telencephalon
TG: Tectal gray
Th: Thalamus
TH: Tyrosine hydroxylase
THy: Terminal hypothalamus
TS: Triangular septal nucleus
Tz: Trapezoid body
VB: Ventrobasal complex
VCo: Ventral cochlear nucleus
VDB: Vertical diagonal band nucleus
VH: Ventral horn
VHO: Periventricular hypothalamic organ
VLL: Ventral nucleus of lateral lemniscus
VMH: Ventromedial hypothalamic nucleus
VNUT: Vesicular nucleotide transporter
VPM: Ventral premamillary nucleus
VTA: Ventral tegmental area
VTg: Ventral tegmental nucleus
ZLO: Zona limitans organ
