# Supplementary figures and images for: Salient brain entities labelled in P2rx7-EGFP reporter mouse embryos include the septum, roof plate glial specializations and circumventricular ependymal organs
Source: Brain Struct Funct. 2021 Jan 11;226(3):715–41. doi: 10.1007/s00429-020-02204-5 (PMC7981336; doi:10.1007/s00429-020-02204-5)

a

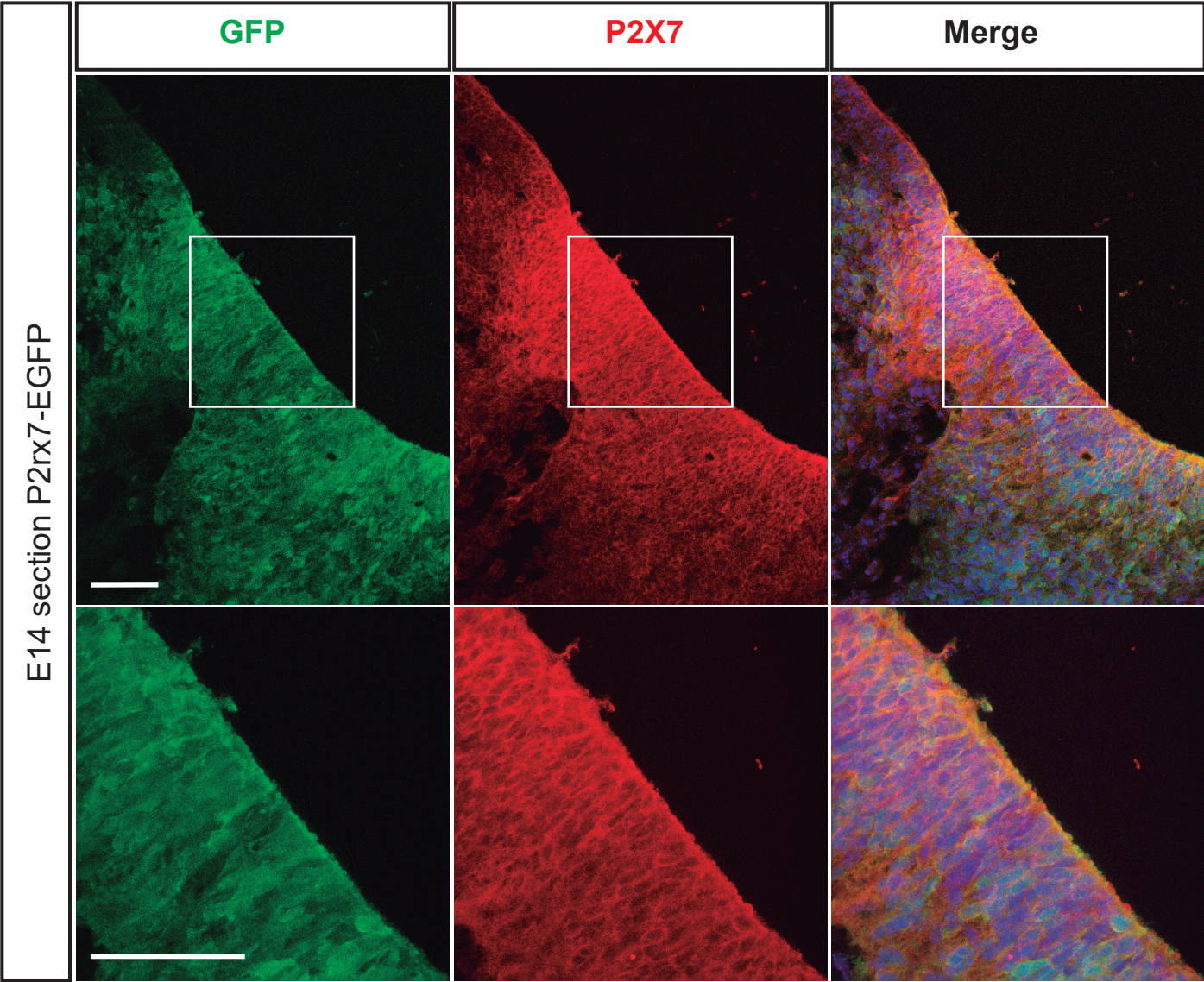

b

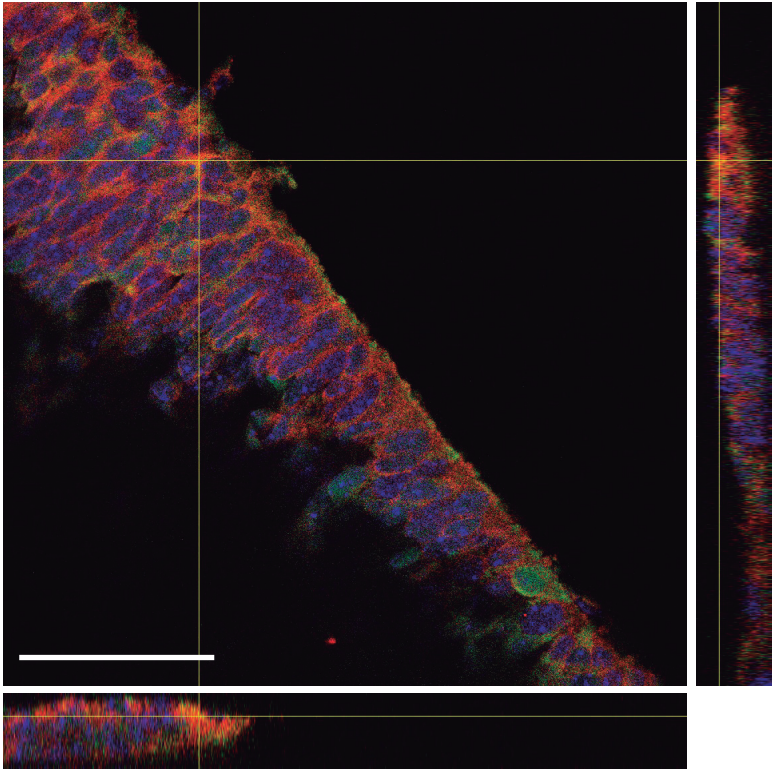

Supplement: Supplementary file 1 — Supplementary Fig.1 Distribution pattern of EGFP reporter and P2X7R in brain sections from P2rx7-EGFP mouse embryos. a Co-labeling of brain slice from E14 embryo with anti-GFP antibody (green), anti-P2X7 receptor antibody (red) and DAPI (blue). Representative results from n = 3 mice are shown. Scale bar: 50 μM, b Orthogonal projection analysis of a magnified subfield from merge image shows the high co-localization of EGFP signal and P2X7R expression (PDF 5257 KB) [file 429_2020_2204_MOESM1_ESM.pdf]
